# Whole-Brain Connectome of GABAergic Neurons in the Mouse Zona Incerta

**DOI:** 10.1007/s12264-022-00930-w

**Published:** 2022-08-19

**Authors:** Yang Yang, Tao Jiang, Xueyan Jia, Jing Yuan, Xiangning Li, Hui Gong

**Affiliations:** 1 Britton Chance Center for Biomedical Photonics, Wuhan National Laboratory for Optoelectronics, MoE Key Laboratory for Biomedical Photonics, Huazhong University of Science and Technology, Wuhan, 430074 China; 2 Research Unit of Multimodal Cross Scale Neural Signal Detection and Imaging, Chinese Academy of Medical Sciences, HUST-Suzhou Institute for Brainsmatics, JITRI, Suzhou, 215123 China

**Keywords:** Zona incerta, GABAergic neurons, Whole-brain connectome, Input circuit, Output circuit, Topological connection

## Abstract

The zona incerta (ZI) is involved in various functions and may serve as an integrative node of the circuits for global behavioral modulation. However, the long-range connectivity of different sectors in the mouse ZI has not been comprehensively mapped. Here, we obtained whole-brain images of the input and output connections *via* fluorescence micro-optical sectioning tomography and viral tracing. The principal regions in the input-output circuits of ZI GABAergic neurons were topologically organized. The 3D distribution of cortical inputs showed rostro-caudal correspondence with different ZI sectors, while the projection fibers from ZI sectors were longitudinally organized in the superior colliculus. Clustering results show that the medial and lateral ZI are two different major functional compartments, and they can be further divided into more subdomains based on projection and input connectivity. This study provides a comprehensive anatomical foundation for understanding how the ZI is involved in integrating different information, conveying motivational states, and modulating global behaviors.

**Supplementary Information:**

The online version contains supplementary material available at 10.1007/s12264-022-00930-w.

## Introduction

The zona incerta (ZI) is a large nucleus in the hypothalamic region with extensive connections throughout the brain [1], and is involved in various functions from locomotion to social behavior, including binge-eating [2], sleeping [3], predatory hunting [4, 5], neuropathic pain [6–8], fear memory [9–11], and investigatory and seeking behavior [12, 13]. It is an important target for treating Parkinson’s disease by deep-brain stimulation [14]. GABAergic cells, the most abundant inhibitory neurons in the mammalian brain [15], are the majority of neurons in the ZI [1, 16]. GABAergic ZI neurons are essential for cortical neuron development [17], shifting the cortical activity patterns [18–20], and gating signal flow [19, 21, 22]. And it is regarded as a potential integrative node for global behavioral modulation [16, 23]. Comprehensive investigation of its functions and global roles is enlightening to the understanding of brain mechanisms.

The ZI is connected with nearly every neural center of the neuro-axis, from cerebral cortex to spinal cord [1], and even receives projections from the retina [24]. Its principal connections are with the cerebral cortex, diencephalon, and brainstem. Traditionally, the ZI of rodents can be loosely divided into different sectors based on cytoarchitecture. The functions of distinct ZI sectors varies greatly while the connections are topologically organized. Cortical neurons that project to the ZI are confined to layer V and the projections are organized in a topological manner [25, 26]. The ZI projects heavily to the dorsal thalamus, while the projections to the hypothalamus are mainly from the rostral ZI [27]. The precise connectivity pattern among distinct sectors of the ZI and thalamus is still unclear. As the main connecting region of the ZI, the midbrain has complex circuits with the ZI, such as superior colliculus (SC)–ZI connections that are organized in a topological manner [28–30]. Since the ZI is a potential integrative node, its connectivity map is enlightening to the understanding of its complex functions and global roles and even brain mechanisms. Although the development of transgenic animals and viral tracing has accelerated the investigation of the input and output connections in interesting ZI sectors [2], there is still a lack of comprehensive comparison of the connection patterns of different ZI sectors.

Here, we aimed at providing a comprehensive long-range input-output connectivity map of GABAergic ZI neurons, and obtain the organization patterns of the ZI. To provide a connectivity map of the entire mouse ZI, we selected four injection sites. We applied Cre-dependent monosynaptic rabies virus and Vgat-ires-Cre line mice to characterize afferent connections of GABAergic neurons in the ZI [31], and adeno-associated viral tracing to label GABAergic efferent axons. By quantifying the numbers of labeled neurons and efferent axons, we constructed a long-range input-output connectivity map of GABAergic ZI neurons and analyzed their connection characteristics.

## Materials and Methods

### Animals

This study was approved by the Animal Experimentation Ethics Committee of Huazhong University of Science and Technology, and all animal experiments were conducted in accordance with relevant guidelines. The C57BL/6J mice used in these experiments were purchased from Beijing Vital River (Beijing). The Vgat-ires-Cre (JAX: 028862) mouse line [32] was purchased from the Jackson Laboratory. All mice were housed in an environment with a 12-h light/dark cycle at 22 ± 1°C and with food and water ad libitum. The study was gender-neutral and 6- to 12-week-old mice were used.

### Surgery and Stereotaxic Injection

The mice were anesthetized by intraperitoneal injection of 1% pentobarbital sodium in 0.9% saline at 0.1 mL/10 g body weight. The mice were mounted into a stereotaxic frame and eye ointment was applied. Holes were drilled above the target brain regions. For anterograde or retrograde experiments, tracers were delivered *via* glass micropipettes using a pressure injection pump at 35 nL/min. After the injections, incisions were sutured, lidocaine hydrochloride gel was applied to the wound, and the mice were returned to their home cages for recovery. Monosynaptic rabies virus was used to label the whole-brain inputs of GABAergic neurons in distinct ZI sectors. 150 nL of a 2:1 mixture of rAAV9-EF1α-DIO-RG-WPRE-pA and rAAV9-EF1α-DIO-DsRed-TVA-WPRE-pA, all AAV viruses with final titers at 2×10^12^ viral genomes (vg)/mL (virus from BrainVTA) of helper-viruses were injected into target ZI sectors. The following coordinates (mm from bregma) were used: rostral ZI (ZIr): AP: −0.95, ML: +0.75, DV: −4.5; intermediomedial ZI (ZIim): AP: −1.95, ML: +1.0, DV: −4.45; intermediolateral ZI (ZIil): AP: −2.15, ML: +1.95, DV: −3.9; caudal ZI (ZIc): AP: −2.55, ML: +2.0, DV: −3.9. Three weeks later, 300 nL RV-△G-EnVA-eGFP (2 × 10^8^ vg/mL, from BrainVTA) were injected into the same sector, and 7 days later, the mice were sacrificed. For axonal AAV-tracing, 100 nL of rAAV2/5-EF1α-DIO-eYFP-WPRE-pA (2 × 10^12^ vg/mL) was injected into a target ZI sector at the above coordinates. Four weeks later, the mice were sacrificed. To verify the projection pattern of ZIim, 50 nL red retrobeads (R180–100, Lumafluor) was injected into the periaqueductal gray (PAG; in mm: AP: −4.0, ML: +0.3, DV: −2.8), and 50 nL fluorescent gold (80014, Biotium) was injected into the SCm (AP: −4.0, ML: +1.5 m, DV: −2.3). Seven days later, the mice were sacrificed. To explore whether single neurons in the ZIr innervated the rostral (AP: −3.6, ML: +0.05, DV: −2.0) and caudal (AP: −4.4, ML: +0.05, DV: −1.8) parts of the SC, 50 nL red retrobeads and 50 nL fluorescent gold were injected into the rostral and caudal parts of the medial SC, respectively, and 7 days later, the mice were sacrificed.

### Histology

Anesthetized mice were perfused with 0.01 mol/L phosphate-buffered saline (PBS, Sigma-Aldrich) and then with 4% paraformaldehyde (PFA, Sigma-Aldrich). The brain was removed from the skull, and post-fixed in 4% PFA at 4℃. Each brain was rinsed 3 times with 0.01 mol/L PBS and embedded in 5% agarose (Sigma-Aldrich). Coronal sections (50 µm) were cut on a vibratome (VT1000, Leica Microsystems). Every second section was used for whole-brain imaging with an automated slide scanner (VS120 Virtual Slide, Olympus).

### Immunostaining

To verify the type of starter cells, we applied immunofluorescent staining to coronal sections near the injection site. After the sections were rinsed 3 times with 0.1M PBS, the sections were blocked with 5% bovine serum albumin in 0.3% Triton X-100 for 1 h at room temperature. Then the sections were incubated overnight with rabbit anti-GABA (1:800, Sigma) at 4℃, then rinsed 3 times with 0.01 mol/L PBS and incubated with goat anti-rabbit 647 (1:500, Thermo Fisher) at room temperature for 2 h. The sections were rinsed 3 times with PBS and then mounted on glass slides. The injection site was imaged by confocal microscopy (LSM 710, Zeiss).

### Embedded Processing

Brains were embedded for whole-brain imaging [33]. After each brain was rinsed 3 times with 0.1 mol/L PBS, it was dehydrated in a graded ethanol series (50%, 70% and 95% ethanol in double distilled water) at 4℃. Then the brains were immersed in a graded glycol methacrylate (GMA) series (70%, 85%, and 100% GMA, with 0.2% Sudan black B in ethanol) at 4℃. The brains were immersed in 100% GMA at 4°C for 3 days. Finally, the samples were embedded.

### Imaging

To obtain the input and output images of the whole brain, we applied dual-channel imaging (output samples were stained with propidium iodide) on the embedded brains [34]. The fluorescence micro-optical sectioning tomography (fMOST) system developed by our group was used to conduct whole-brain imaging at a resolution of 0.32 μm × 0.32 μm × 2 μm.

### Data Processing

We used a homemade method to count the numbers of neurons and fiber signals [35]. NeuroGPS was used to obtain the coordinates of the neuron somata. To distinguish the projection signal from background, we used a Gaussian filter to remove the background and images were binarized. Easily distinguishable regions were segmented to obtain the conversion parameters for transforming the whole image stack to Allen CCFv3. All results were checked manually to eliminate errors. To quantify the input and output connections, we transformed the coordinates of the somata of input neurons and image stacks of labeled signals to Allien CCFv3. Then the number of somata or the volume of the projection signal in each area were quantified. Regions with a connection strength of <0.03% were set to 0, and the ZI was set to 0. We merged some areas with low connection strength for ease of display. To unify the connection strength between the different samples, we calculated the percentage of connections in different regions.

### Visualization and Statistical Analysis

Amira (v6.1.1, FEI), ImageJ, and Python 3.8.4 were used to visualize the input and output results. To compare the differences in connection strength between different brain regions, we used one-way ANOVA. Pearson correlation coefficients were used to assess the similarity of connections. Hierarchical cluster analysis and correlation analysis were used to analyze the similarities and variances of the connection strength between different samples or brain regions. These analyses were performed using MatLab (v2017a, MathWorks) and Python 3.8.4. All histograms, scatter diagrams and heat maps were generated by GraphPad Prism (v.6.0, GraphPad) and Python 3.8.4.

## Results

### Experimental Strategy to Reveal the Connectivity of Distinct ZI Sectors

**Fig. 1:**
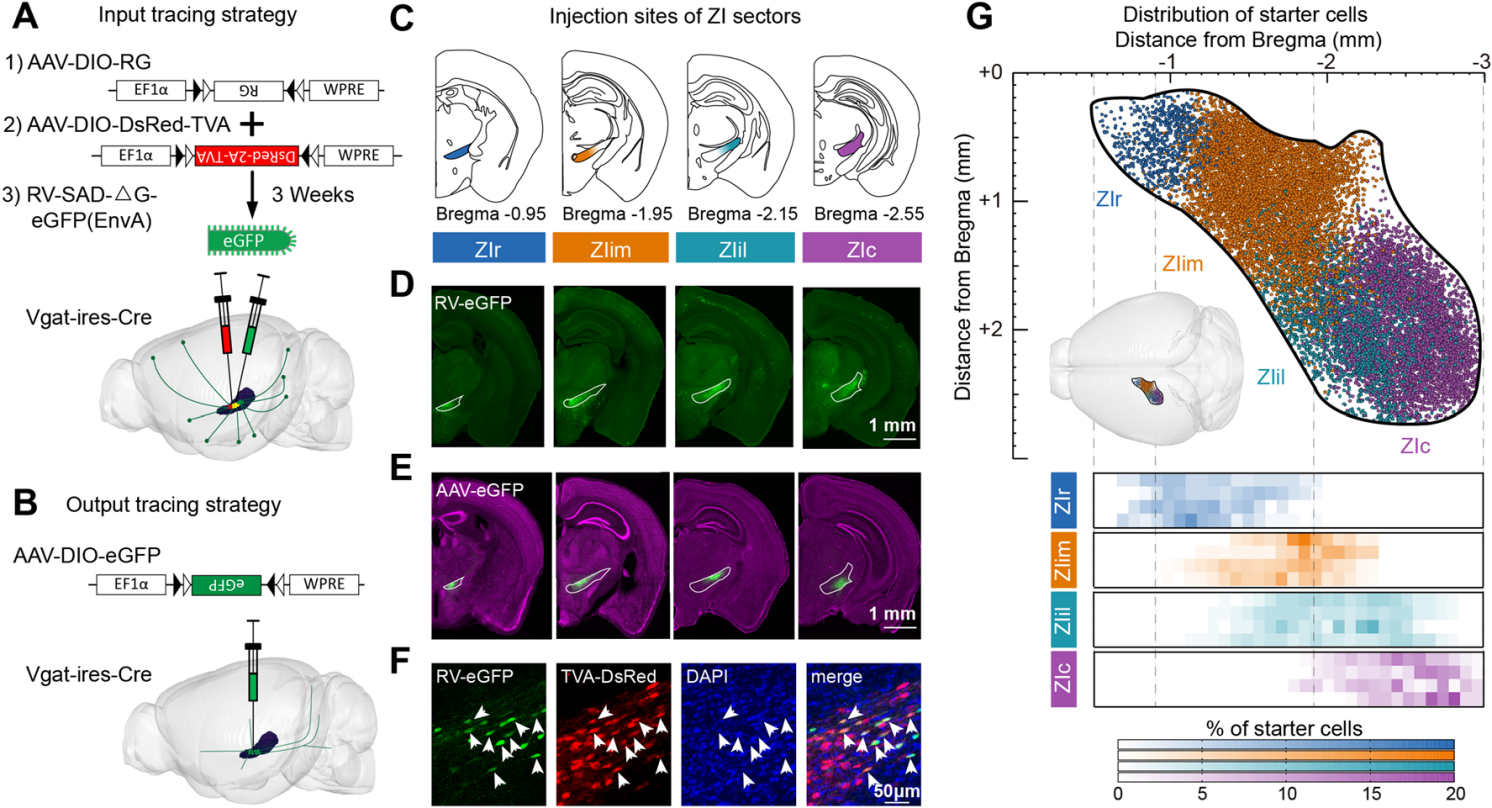
Whole-brain tracing of input and projections of distinct ZI sectors. **A** Schematic of the monosynaptic retrograde tracing strategy. For retrograde tracing, AAV9-EF1α-DIO-RG (1) and AAV9-EF1α-DIO-DsRed-TVA (2) are co-injected into the ZI sector of interest. Three weeks later, RV-SAD-△G-eGFP (EnvA) (3) is injected into the same sector. Only with the help of TVA and RG can RV-eGFP spread retrogradely to presynaptic neurons. **B** Schematic of the anterograde tracing strategy. AAV2/5-DIO-eGFP is injected into the ZI sector of interest, and the axons of infected GABAergic neuron are labeled with eGFP. **C** Locations of distinct ZI sectors in this experiment: ZIr (blue), ZIim (orange), ZIil (cyan), and ZIc (purple). **D** RV-eGFP expression near the injection sites in coronal sections (see Fig. S2A for distribution of starter cells). **E** Coronal sections showing AAV-eGFP expression in the injection sites. **F** Identification of starter cells. Cells co-expressing AAV-DsRed and RV-eGFP are considered to be starter cells. Starter cells of ZIim (see Fig. S2B). Scale bars, 1 mm (**D**), 1 mm (**E**), and 50 μm (**F**). **G** Schematic of the dorsal view of retrograde tracing starter cells in distinct ZI sectors and heat map showing the starter cell distribution in each sample at each specific ZI target (lower panels). Center left insert, visual aid representation the location of ZI in the brain. Every row in each ZI sector is from one sample, *n* = 4 mice per condition in retrograde strategy.

The ZI is a large elongated string-like structure located in the lateral hypothalamus. To map the connectivity of the entire mouse ZI, we selected four injection sites, and viral tracers were injected into each of these regions. The four sectors were named rostral, intermediomedial, intermediolateral, and caudal (ZIr, ZIim, ZIil, and ZIc; see Fig. 1C).

To label the GABAergic neurons, we used transgenic mice and cre-dependent viral tools. Vgat-ires-Cre mice, which expressed Cre recombinase directed to GABAergic neuronal cell bodies [32], were used to obtain the whole-brain connectivity map of GABAergic ZI neurons. To demonstrate the monosynaptic inputs of the ZI, we injected two Cre-dependent adeno-associated viruses (AAV9-EF1α-DIO-DsRed-TVA and AAV9-EF1α-DIO-RG) into the ZI at the same time. Three weeks later, RV-SAD-△G-eGFP (EnvA) was injected into the same ZI sector (Fig. 1A). The TVA-expressing cells infected with the modified, high specificity, rabies virus (RV), and labeled the presynaptic neurons only with the help of Rabies glycoprotein G (RG). The reliability of the experimental strategy was confirmed by control experiments (Fig. S1). To trace and quantify the efferent axons (outputs) of the ZI, we injected Cre-inducible adeno-associated virus (AAV2/5-EF1α-DIO-eGFP) into the ZI, and GABAergic neurons infected by AAV virus were labeled with enhanced green fluorescent protein (eGFP) (Fig. 1B). To comprehensively analyze the whole-brain connectivity at single-cell resolution, we obtained the inputome and projectome datasets with fluorescent micro-optical sectioning tomography (fMOST) [34]. As shown in Fig. S2B, the virus-labeled mouse brains were perfused and embedded in resin, then were imaged with fMOST. The datasets were processed and quantified as in our previous reports [35].

To confirm the performance of the virus, we checked coronal sections from monosynaptic retrograde tracing samples, and found that nearly every neural center of the neuraxis had eGFP-positive cells (Fig. S3A). The highest density of eGFP-positive neurons was near the injection sites (Fig. 1D). The coronal sections of anterograde tracing samples were checked too (Fig. S3B). We found that eGFP-positive efferent axons were present in many brain regions, such as the thalamus, hypothalamus, and brainstem. The cell bodies were confined to the ZI (Fig. 1E).

In monosynaptic retrograde tracing experiments, cells co-expressing DsRed and eGFP were counted as starter cells (Fig. 1F). We only analyzed the data with >80% of starter cells located in the ZI to ensure accuracy (Fig. S2D). A few starter cells were located outside of ZI, including in the lateral hypothalamic area (LHA), posterior hypothalamic area (PH), and reticular thalamic nucleus. To compare among distinct ZI sectors, the location of starter cell was certified in the continuous dataset. Most of the starter cells of ZIr were distributed at −0.7 to −1.5 from bregma, while most of those of ZIim, ZIil and ZIc were distributed at −1.5 to −2.2, −1.6 to −2.5, and −2.1 to −2.7 from bregma, respectively (Fig. 1G). Most starter cells of ZIim were located in the medial ZI, and most starter cells of ZIil were located in the lateral ZI. In tracing experiments, we described the distribution of fluorescence (Fig. S2E) and found a similar distribution. Starter cells almost covered the entire ZI in both RV and AAV tracing, and partial regions of distinct ZI sectors were overlapped. Our subsequent analysis also found that the connectivity patterns of distinct ZI sectors showed significant differences. These data showed that viral labelling samples can reflect the characteristics and variation rules of connectivity of distinct ZI sectors.

To quantitatively analyze the whole-brain input-output connectivity maps, the number of eGFP-labeled cells and the fluorescent signal of efferent fibers were calculated (see Materials and Methods for details).

### Whole-brain Mapping of the Input/Output

The ZI has widespread connectivity with nearly all neural centers. Here, we analyzed the connections among the ZI and 145 anatomical brain regions (details in Figs S4, S5). Although connecting brain regions were distributed throughout almost the whole brain, the main connections were concentrated in a few regions. The ZI received inputs from 126 regions, and half of the inputs were from ~10 regions (9, 13, 11, and 8 regions in ZIr, ZIim, ZIil, and ZIc respectively; Fig. S6A). While its efferent fibers were distributed in 77 regions and half of them were in ~5 regions (5, 4, 5, and 4 regions in ZIr, ZIim, ZIil, and ZIc respectively; Fig. S6B). These results indicated that the number of output regions was less than that of input regions. To overview the whole-brain connectivity, the input-output regions were divided into 15 larger regions.

**Fig. 2:**
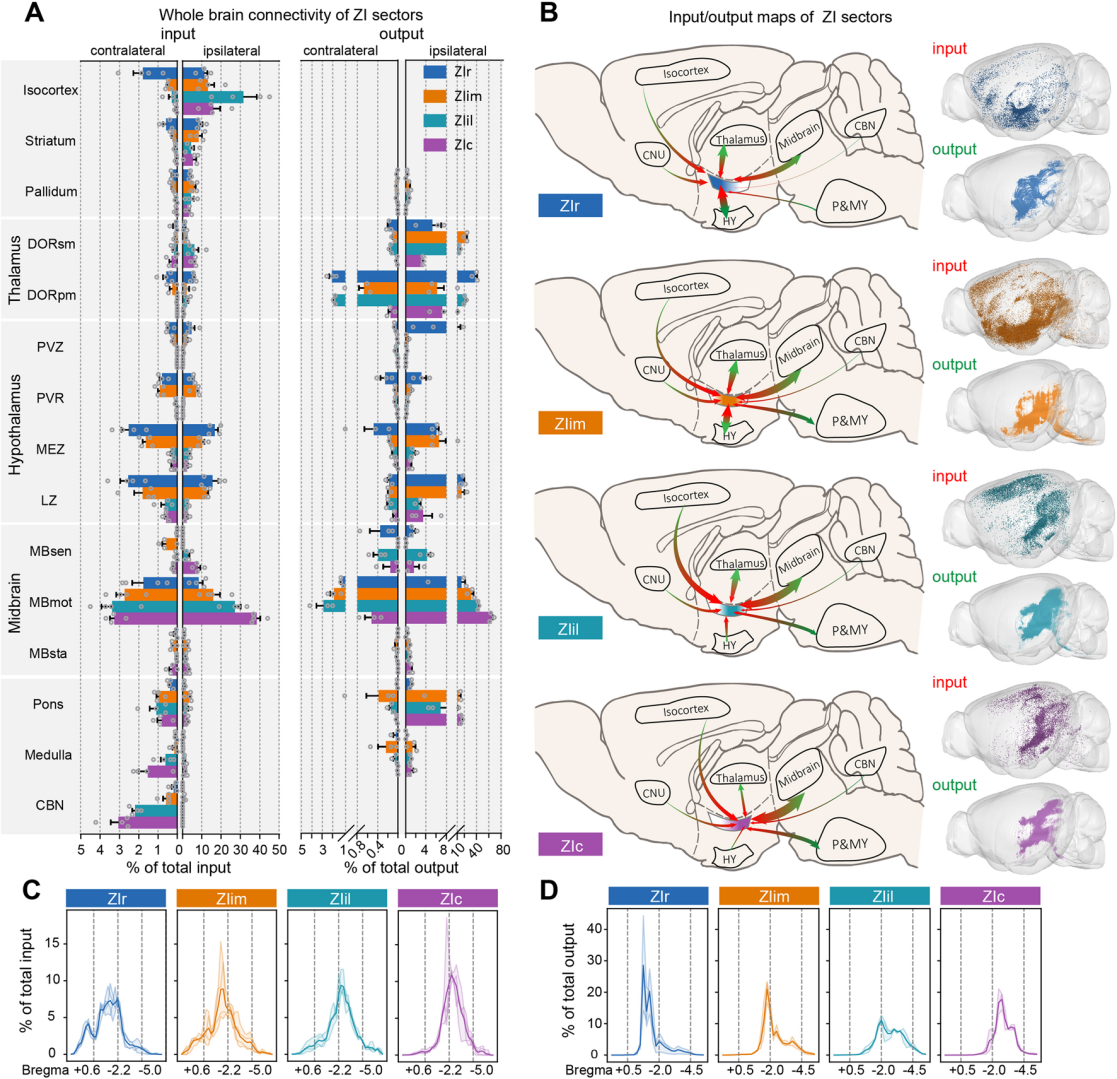
Overview of ZI connectivity. **A** Whole-brain connectivity of ZI sectors. Ipsilateral and contralateral inputs (left) and outputs (right) of the four ZI sectors. Data are shown as the mean ± SEM. **B** Individual input-output connectivity patterns of the ZI sectors. Left, schematics of the input-output connection patterns of major brain regions (green arrowheads, outputs; red arrowheads, inputs; area of arrowhead, strength of connection). Right, three-dimensional visualization of whole-brain inputs to and outputs from the ZI in representative samples. **C** Percentages of inputs plotted along the rostro-caudal axis. **D** Percentages of outputs plotted along the rostro-caudal axis. Bin width, 200 μm. Thick lines, mean; thin lines, individual animals. *n* = 4 mice per condition in (**A**) (left, input data) and (**B**). *n* = 3 mice per condition in (**A**) (right, output data) and (**D**). Colors indicate data from each ZI sector: ZIr, blue; ZIim, orange; ZIil, cyan; ZIc, purple.

We compared the input and output of these regions, and generated connection maps for ZI sectors (Fig. 2A, B). The principal connections of the ZI were isocortex, thalamus, hypothalamus, and midbrain. There were differences between the input and output brain regions. ZI neurons received abundant input from the cortex and striatum, but few efferent fibers projected back to these regions. Although there were labeled fibers in isocortex and striatum, their ratio in the whole output circuit was very low (<0.03%). The ZI also received input from the cerebellar nuclei (CBN), but no labeled fibers were observed in these nuclei. In the remaining regions with interconnections, the output intensity was generally higher than the input, except for the pallidum (input, 4.61% ± 1.91%; output, 0.41% ± 0.32%; ***P = 9.89 × 10^−8^). In general, GABAergic ZI neurons received more inputs from the telencephalon cerebrum than projected back.

To furtherly compare the ZI circuit with different brain regions, we analyzed the ipsilateral and contralateral connectivity (Fig. 2A). All ZI sectors basically received bilateral afferent connectivity from the input regions, and the contralateral input was generally a tenth of that on the ipsilateral (Fig. S6C). The ZI received a higher proportion afferent connectivity from the contralateral pons and medulla. The ZI mainly received input from the contralateral CBN, while eGFP-positive neurons were found in the ipsilateral CBN. Different from the input, the ZI mainly sent efferent fibers to ipsilateral regions, and the ipsilateral output was generally >10 times the contralateral projections (Figs 2A, S6D). In a few regions, the ratios of ipsilateral to contralateral connection were different. For example, the proportion of input from the contralateral isocortex decreased as the injection site was moved backward along the rostro-caudal axis (Figs S6C, S7A).

While there were some quantitative differences, we compared the connections of the four ZI sectors and found that the connections of these regions changed gradually. In general, as the injection site was moved backward along the rostro-caudal axis, the connection centers gradually moved backward too (Fig. 2C, D), and several regions also showed a similar tendency. As the ZI sector was moved backward, the input from motor-related midbrain (MBmot) increased (10.21% ± 4.44%, 18.57% ± 6.00%, 30.52% ± 5.92%, and 41.69% ± 3.43% to ZIr, ZIim, ZIil, and ZIc, respectively). Meanwhile, the efferent fibers from ZI to MBmot also had a same varying tendency (15.95% ± 8.07%, 32.72% ± 4.88%, 45.39% ± 1.77% and 65.00% ± 3.30% from ZIr, ZIim, ZIil and ZIc, respectively). The connectivity of some remaining regions showed a similar tendency, such as input from the CBN (0.19% ± 0.17%, 1.28% ± 0.54%, 2.19% ± 0.24%, and 3.08% ± 0.68% to ZIr, ZIim, ZIil, and ZIc, respectively). Meanwhile, the afferent connectivity from the hypothalamus showed preference too, in that ZIr and ZIim received a large amount but ZIil and ZIc only received a little. Afferent connectivity from the medial zone of the hypothalamus to the ZIr was significantly stronger than that to the ZIim (*P* = 0.0188). Similar to the input, the efferent fibers from ZIr and ZIim to the hypothalamus were much more numerous than those from ZIil and ZIc. The pons, medulla, and CBN also had connections with the entire ZI, while mainly connecting with ZIil and ZIc. These preferred connections were an important source of the circuit differences of ZI sectors (Fig. S6C, D).

Here, we analyzed the connectivity of ZI from the whole-brain perspective, found the principal regions connected with the ZI, and analyzed the tendency of connectivity to vary. However, there were major differences in the connection patterns. To further demonstrate the organizational characteristics of ZI sectors, we showed their detailed connections with the isocortex, diencephalon, and midbrain.

### Connections Between ZI and Isocortex are Topological

The ZI received projections from most areas of cortex, and the connections were organized topographically. The topology of connection is helpful to understand the functions of ZI. Here, we summarize the projection pattern of the cortical regions to ZI, and further discuss the way cortical input regions change.

**Fig. 3:**
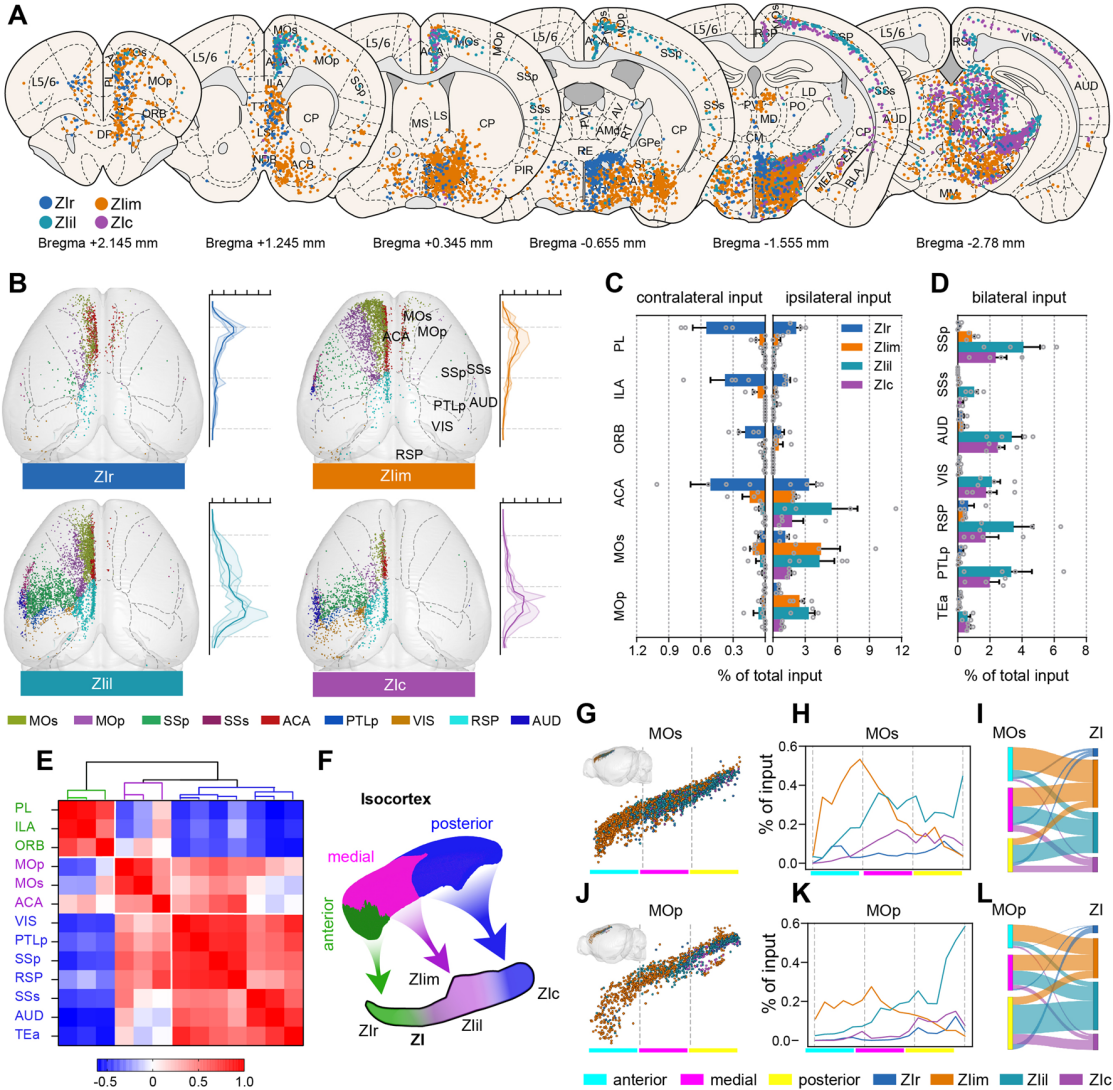
Isocortex-ZI connectivity. **A** Schematic coronal sections depicting the distribution of eGFP-positive cells (slice thickness, 100 μm). **B** Two-dimensional representation of the isocortex inputs to ZI sectors. Curves on the right corresponds to the isocortical input strength along the rostro-caudal axis (bin width, 200 μm; thick lines, mean; thin lines, individual animals). **C** Ipsilateral (right) and contralateral cortical inputs (left) to the four ZI sectors. **D** Bilateral cortical inputs to the four ZI sectors. **E** Heat map of Pearson’s correlation coefficient matrix and hierarchical cluster analysis showing that the projections from cortex to ZI can be roughly divided into three categories: anterior, medial, and posterior (pair-wise correlations on the data organized into 16 input samples). **F** Schematic of the connection patterns between cortex and ZI sectors. **G**, **J** Lateral view of the distribution of retrogradely-labeled eGFP-positive neurons from distinct ZI sectors in MOs and MOp (upper left inserts, visual aid representation of the location of MOs or MOp). **H**, **K** Distribution of labeled neurons in MOs and MOp varies with the rostro-caudal axis (bin width, 200 μm; data are presented as the mean; MOs and MOp divided into anterior, middle, and posterior parts). **I**, **L** Schematic of MOs and MOp projections to the ZI (width of the curve represents input strength). The same data sets of representative input samples are used in **A**, **B**, **G,** and **J**. *n* = 4 mice per condition in **C**, **D**, **E**, **H,** and **K**. Each dot represents one RV-labeled cell in **A**, **B**, **G,** and **J**. Colors indicate data from each ZI sector: ZIr, blue; ZIim, orange; ZIil, cyan; ZIc, purple.

The ZI received projections from most regions of the isocortex, and the principal connections were sensory, motor and association cortices (Fig. 3A). We found that the eGFP-positive neurons resided in lamina V of the cortex. All ZI sectors received similar strength of input from cortex (one-way ANOVAs showed no significant difference between the four ZI sectors, except for ZIr *vs* ZIil, *P* = 0.0421; Fig. S6E), but the eGFP-positive cells in the cortex varied widely (Fig. 3A, B). As the ZI injection sites were moved backward, the connection centers in isocortex moved backward (Fig. 3B). To confirm the source of the difference, we quantitatively compared the main cortical inputs (Fig. 3C, D). We found that the input regions varied greatly, and the proportion of ipsilateral cortical input gradually increased (ratios of ipsilateral isocortical input to whole isocortical inputs: ZIr, 87.0% ± 1.7%; ZIim, 95.8% ± 1.1%; ZIil, 99.3% ± 0.3%; ZIc, 99.3% ± 0.4%; Fig. S7A). ZIr received the most input from the contralateral cortex, while both ZIil and ZIc received inputs almost entirely from the ipsilateral cortex.

The projections patterns of cortical regions to ZI sectors can be roughly divided into three categories. The first is composed of the bilateral prelimbic, infralimbic, and orbital areas. These regions mainly projected to the ZIr and ZIim, but rarely to ZIil or ZIc. The second category contained the bilateral anterior cingulate, secondary motor (MOs) and primary motor areas (MOp). These regions mainly projected to ZIim and ZIil, and had weaker projections to ZIr and ZIc. Although the input of the second category was also bilateral, the ratio of ipsilateral isocortical input to whole cortical inputs decreased. The last category consisted of remaining cortical regions, which mainly projected to the ipsilateral ZIil and ZIc. To determine whether this classification was reasonable, we used correlation and hierarchical cluster analysis of the cortical inputs (Fig. 3E, F) and this showed that the input regions could be roughly divided into three categories.

The spatial differences in the distribution of cortical eGFP-positive neurons (Fig. 3D) showed topological inputs from cortex to ZI, while the input strength from MOs and MOp did not significantly differ in ZIim and ZIil (*P* >0.05; Fig. S7B). To determine whether the gradual change in the nature of the input patterns also occurred in these regions, we divided them into anterior, middle, and posterior parts (Fig. 3G, J). Although all parts of the MOs and MOp had afferent connectivity to distinct ZI sectors, these regions preferentially targeted different sectors of the ZI (Fig. 3H, K). These findings suggested that ZIr mainly received input from the posterior part of MOp, while anterior and middle MOs and MOp projected to the ZIim, middle and posterior MOs to the ZIil, and middle and posterior MOs as well as posterior MOp to the ZIc (Fig. 3I, L). Here, we verified that the change in input patterns was gradual, and defined the connection patterns between ZI and motor cortex. These result improve the understanding of the roles of distinct parts of the ZI in motor functions.

### Connections Between ZI and Diencephalon are Topological

As a part of the lateral hypothalamus, the ZI is located at the junction of the hypothalamus and thalamus, and has strong interconnections with the diencephalon, so we investigated the characteristics of these connections.

**Fig. 4:**
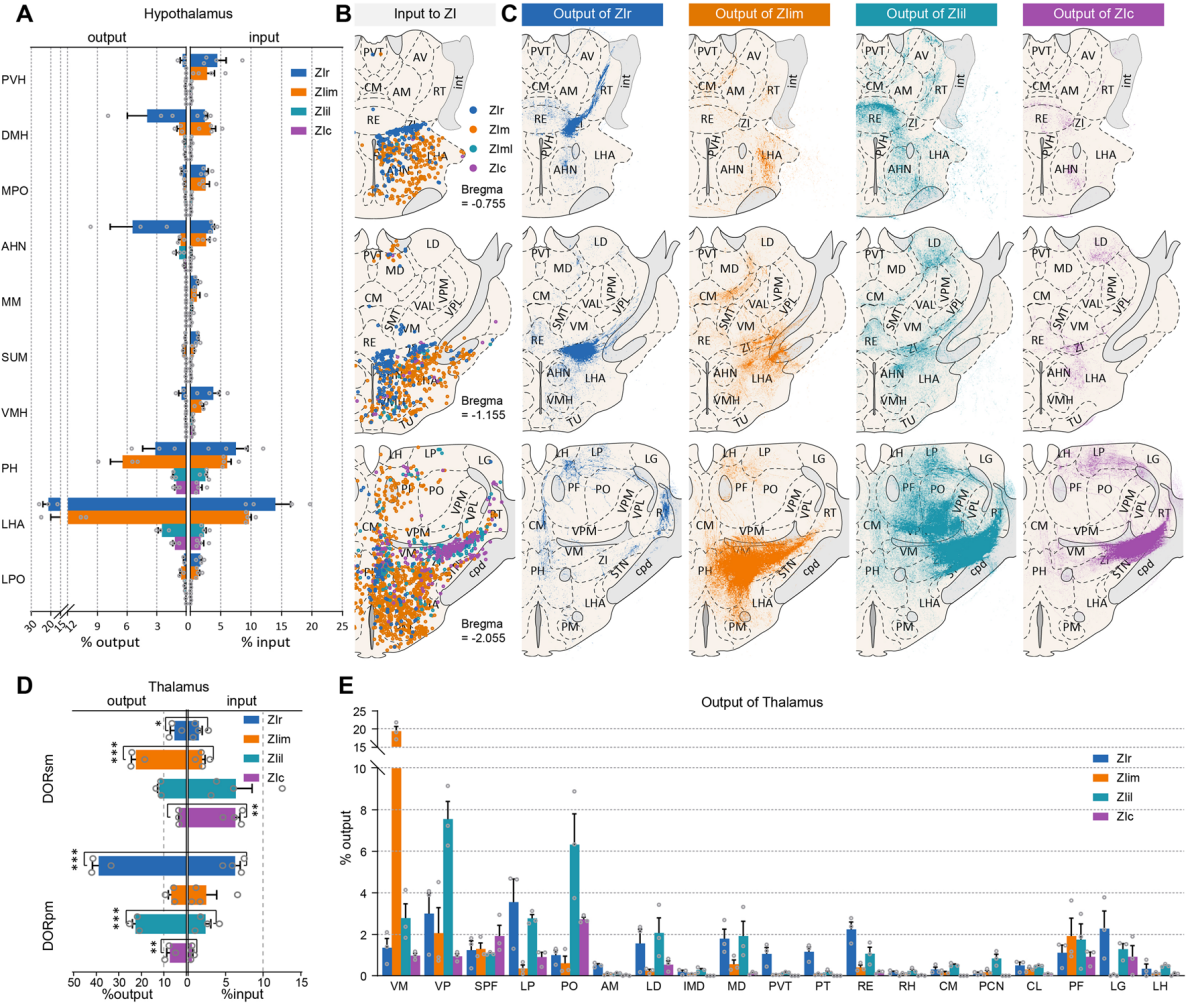
ZI-diencephalon connectivity. **A** Outputs from ZI to the hypothalamus (left) and inputs detected in the hypothalamus (right). **B** Schematic coronal sections depicting the distribution of eGFP-positive cells. Each dot represents one RV-labeled cell. **C** Schematic coronal sections depicting the distribution of eGFP. The eGFP signals represent the output of ZI sectors. **D** Outputs from ZI to the thalamus (left) and inputs detected in the thalamus (right). **E** Outputs from the four ZI sectors to the thalamus (mean ± SEM; ***P <0.001, **P <0.01, *P <0.05. Brain slice thickness is 100 μm in **B** and **C**. *n* = 3 mice per condition in **A** (left, output data), **D** (left, output data), and **E**. *n* = 4 mice per condition in **A** (right, input data) and **D** (right, input data). Colors indicated data from each ZI sector: ZIr, blue; ZIim, orange; ZIil, cyan; ZIc, purple.

The hypothalamus had stronger interconnections with ZIr and ZIim than with ZIil and ZIc (Fig. 4A). ZIr and ZIim had strong interconnections with the PH and LHA, as did both ZIil and ZIc, the latter being rarely mentioned in previous studies (Fig. 4A–C). The connections between ZIr and ZIim did not significantly differ for the hypothalamus, except for the supramammillary nucleus (SUM) (Fig. S8A, B). The afferent connectivity from SUM to ZIr was stronger (*P* = 0.0133) than that to ZIim, while the innervation from ZIil to SUM was stronger (*P* = 0.0037) than that from ZIr. We also observed that in some coronal sections ZIr innervated the medial hypothalamus, and ZIim tended to innervate the lateral hypothalamus (Figs. 4C and S8C, D), but a difference did not show up in the quantitative results.

We found that ZI sent rich efferent fibers to thalamus, but the eGFP-positive cells were only rich in few brain regions (Fig. 4B, C). By comparing the input and output, we found that outputs of the sensory-motor cortex-related thalamus and polymodal association cortex-related thalamus were generally stronger than their inputs (Fig. 4D). The thalamic connections among distinct ZI sectors were significantly different, and the connections were also topological (Fig. S8E). The paraventricular nucleus of the thalamus had a strong bidirectional connection with ZIr, and the connection strength was higher than for other ZI sectors (input, *P* <0.01; output, *P* <0.05. Figures 4E, S8E). The lateral habenula projected heavily to ZIr and ZIim, and the afferent connectivity of ZIim was stronger than that to ZIil and ZIc (*P* <0.05). Specifically, the ventral medial nucleus of the thalamus (VM) was the main output region of ZIim, while the efferents to the VM were stronger than other ZI sectors (*P* <0.001). The subparafascicular nucleus (SPF), peripeduncular nucleus (PP), and lateral geniculate complex (LG) were the main afferent connectivity sources of ZIil and ZIc, and the efferent fibers of these sectors to the SPF and PP were stronger than those of ZIr and ZIim (*P* <0.05). Surprisingly, compared with ZIim, ZIr projected more axons to the LG (ZIr, 2.38% ± 1.08%; ZIim, 0.08% ± 0.04%). The posterior complex of the thalamus (PO) mainly received innervation from ZIil and ZIc, and that was significantly stronger than ZIr and ZIim (*P* <0.05). Efferent fibers from the four ZI sectors were detected in the parafascicular nucleus (PF) and no statistically significant difference was found (*P* >0.05). But we found that a spatial difference, in that ZIil fibers tended to be distributed in the ventral PF, while the fibers of other ZI sectors mainly projected to the dorsal PF (Fig. 4C). In addition, the projection from the ZI to the thalamus was highly topologically organized. For example, neurons projecting to the ventral or dorsal part of the PO may be two groups of neurons that are distributed differently in the ZI (Fig. S8F–K).

In general, the connection between the ZI and parts of the diencephalon is topological. We found that, in some coronal sections, ZIr innervated the medial hypothalamus, and ZIim tended to innervate the lateral hypothalamus. The projections of distinct ZI sectors to the thalamus not only differ in the strength of connectivity, but also in regional connectivity.

### Connections Between ZI and Midbrain are Topological

The midbrain had strong interconnection with different ZI sectors and the strength increased as the injection sites moved backward along the rostro-caudal axis. Here, we investigated this phenomenon and the topological projections to the SC and PAG.

**Fig. 5:**
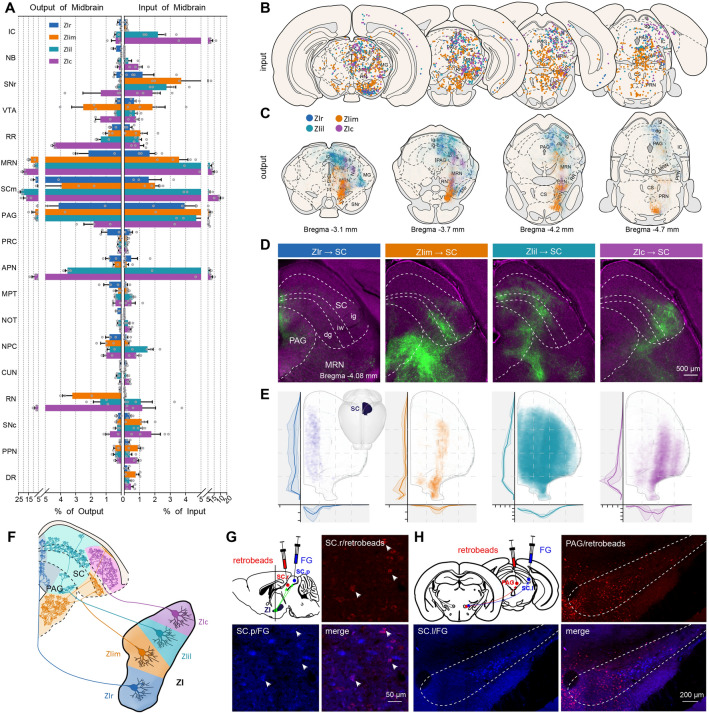
ZI- midbrain connectivity. **A** ZI projections to the midbrain (left) and inputs from the midbrain (right). **B** Schematic coronal sections depicting the distribution of eGFP-positive cells. Each dot represents one RV-labeled cell. **C** Schematic coronal sections depicting the distribution of projection fibers. **D** Representative images of projections from four ZI sectors to the midbrain. Scale bar, 500 μm. **E** Two-dimensional representation of the projection fibers in the superior colliculus from the four ZI sectors (upper left, distribution of the eGFP signal in SC along the rostro-caudal axis; lower curves, distribution of projection fibers in the SC along the medial-lateral axis; left insert, visual aid representation the location of the SC; thick lines, mean; thin lines, individual animals). **F** Schematic of ZI sectors projecting to the SC and PAG. **G** Single ZI neurons simultaneously projecting to the anteromedial and posteromedial SC. Retrobeads (red) are injected into the anteromedial SC, and fluorescent gold (FG, blue) is injected into the posteromedial C. Some neurons in the rostral ZI are co-labeled by retrobeads and FG. Scale bar, 50 μm. **H** Fibers in the PAG and SC from two different groups of neurons. Retrobeads are injected into the PAG and FG is injected into the SC. The labeled neurons are divided into two spatially separate groups. Scale bar, 200 μm. Data shown as the mean ± SEM. Slice thickness is 100 μm in **B**, **C**, **G**, and **H**. *n* = 3 mice per condition in **A** (left, output data). *n* = 4 mice per condition in **A** (right, input data) and **C**.

To determine whether the phenomenon was caused by differences in the regional connectivity or because the main input regions had similar properties, we compared the inputs and outputs of the midbrain (Fig. 5A). All ZI sectors had a heavy interconnection with the midbrain reticular nucleus (MRN), motor-related SC (SCm), PAG, and anterior pretectal nucleus (APN) (Fig. 5A–C). Some regions exhibited increasing connection strength as the injection site moved backward, while other nuclei of the midbrain mainly connected with ZIil and ZIc. Interconnections between ZIr and the MRN were weaker than other ZI sectors (input, *P* <0.05; output, *P* <0.01). The connections of the SCm with ZIr and ZIim were weaker than those of ZIil and ZIc (*P* <0.05), and the APN also had a heavier connection with ZIil and ZIc (*P* <0.05) (Fig. S9A, B).

When comparing the connections of distinct ZI sectors, we found that the efferent fibers in the SC were topologically organized as previously reported, and the distribution of fibers in the PAG also had a certain topology (Fig. 5C, D). We found that the ZIr mainly targeted the medial SCm and dorsomedial PAG while the ZIim preferred the ventral PAG and ventrolateral SCm, the ZIil mainly projected to the intermediate SCm and lateral PAG, while the ZIil focused on the dorsolateral SCm with a few fibers in lateral PAG. In order to further explore the projection patterns from ZI to SC, we investigated the distribution of efferent fibers in the SCm (Fig. 5E). We describe the distribution of fibers along the rostro-caudal axis and medial-lateral axis in the SCm. The topological projection from ZI to SCm was verified, and we found that the projections had a longitudinal-zonal organization. We defined the topological organization of ZI to SC and PAG projections (Fig. 5F).

As shown in Figure 5D, we found that the efferent fibers from any ZI sector, extended to almost the entire SC along the rostro-caudal axis. To determine whether single neurons in the ZI innervated rostral and caudal parts of SC simultaneously, red retrobeads were injected into the rostral part and fluorescent gold (FG) into the caudal part of the medial SC (Fig. 5G). We found that some neurons were co-labeled with retrobeads and FG, thus individual neurons do simultaneously innervate the rostral and caudal parts of the SC. Many of the efferent fibers to the SCm extended to the PAG and the distribution of efferent fibers in PAG and SCm had a roughly corresponding relationship. To explore whether single neurons in the ZI innervated the SCm and PAG simultaneously or separatey, we injected red retrobeads and FG into the PAG and SCm in individual mice (Fig. 5H, see Fig. S9C for injection sites). Most of the labeled neurons were divided into two spatially separate groups. Neurons that projected to the ventrolateral PAG were located in the intermediomedial ZI, and neurons that projected to the lateral SC were located in central part of the ZI. Thus, neurons of the ZIim innervate PAG and SCm separately.

### Comparison of Input and Output Distributions

**Fig. 6:**
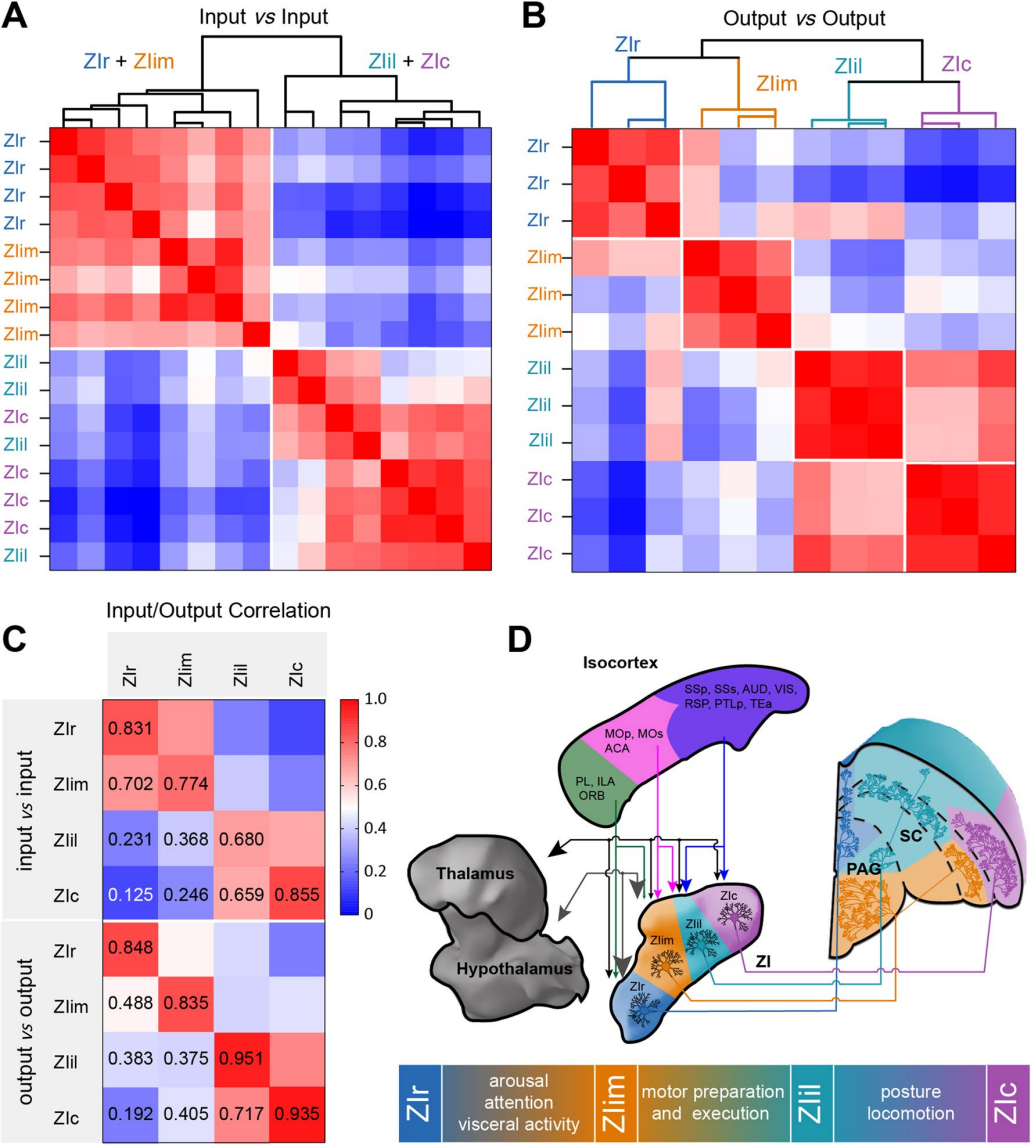
Connectivity characteristics of ZI. **A** Correlation and hierarchical cluster analysis of the inputs (*n* = 16) of the ZI. The pair-wise correlations were calculated for data organized into 126 input regions. **B** Correlation and hierarchical cluster analysis of the outputs (*n* = 12) of the ZI. The pair-wise correlations were calculated for data organized into 77 output regions. The heat maps in **A** and **B** represent Pearson’s correlation coefficient matrixes. Note for both input and output correlations, 2 clusters form from the ZIr/ZIim animals and ZIil/ZIc animals. **C** Correlation coefficients of input (*n* = 4 per condition) and output (*n* = 3 per condition). **D** Schematic of ZI connectivity characteristics. Lower panel, possible functions of distinct ZI sectors. Similar to the connection results, functions may shift in a progressive manner among different sectors. Area of arrowhead indicates strength of connection.

To further investigate whether the distinction among these ZI sectors was meaningful, we compared the correlation coefficients of input or output in a pairwise fashion. The correlation coefficients of inputs and outputs were low, but the correlation coefficients between the outputs of four ZI sectors were slightly higher than the inputs (average correlation coefficient: input, 0.4677 ± 0.2736; output, 0.5111 ± 0.2579). We also hierarchically clustered the input and output regions separately. With both inputs and outputs, two distinct clusters were formed, separating a grouped ZIr/ZIim pool from a grouped ZIil/ZIc pool. The input correlations of ZIr and ZIim tracing were too similar to classify them into separate clusters (average correlation coefficient: 0.7019 ± 0.0777, Fig. 6A, C). For the same reason, we could not divide the inputs of ZIil and ZIc into separate clusters (average correlation coefficient: 0.6590 ± 0.1650, Fig. 6A, C). Although the output correlations of tracing the four ZI sectors were also similar, they fell into four separate clusters (average correlation coefficients: ZIr *vs* ZIim 0.4882 ± 0.1352, ZIil *vs* ZIc 0.7165 ± 0.0911, Fig. 6B, C). Comparing the connection strength between ZIr and ZIim, we found that 31.03% (27/87) of the input areas had significant differences, and 58.33% (28/48) of the output regions had significant differences (Fig. S10A, C). Comparing the connection strength between ZIil and ZIc, we found a significant difference in 29.63% (24/81) of the input regions and 64.44% (29/45) of the output regions (Fig. S10B, D). These results were consistent with the results of hierarchical clustering. The connectivity patterns of ZIr and ZIim were similar, as were those of ZIil and ZIc. The similarity of the inputs was higher than that of the outputs.

## Discussion

Here, we systematically mapped the input-output connectivity of GABAergic ZI neurons and compared the connectivity of four ZI sectors in the whole brain. From the continuous 3D datasets, we comprehensively mapped the ZI connectivity characteristics (Fig. 6D). The connections of ZI were heterogeneous and topologically organized. Cortical input regions can be divided into three categories. The topology of the connections between ZI and the diencephalon was not only manifested in strength, but also in regions. And the efferent fibers in the SC were longitudinally organized. Clustering results showed that the medial and lateral ZI were two different major functional compartments, and they can be further divided into more subdomains based on output connectivity.

Cortex was the main input region of ZI, and it was also an important source of input differences. We then investigated the connections between ZI and cortex more comprehensively. The cortical projection to ZI could be divided into three categories. These were consistent with the results of previous studies with lentivirus showing that the cortical input of the ZI could be divided into sensory, motor, and anterior limbic cortex [25]. The differences in connectivity may underlie the functional differences. First, prefrontal cortex preferentially projected to the ZIr and ZIim. The projection of the medial prefrontal cortex to ZIr bidirectionally regulates the escape speed of mice [16], and the projection of the prefrontal cortex to ZIim is associated with curiosity in mice [12]. This indicated that emotion and cognition may regulate the behaviors of mice through ZIr and ZIim. Second, the motor cortex (MO) projected primarily to the medial ZI, suggesting that these ZI sectors are closely involved in motor planning and execution. But the functions of ZIim and ZIil are quite different. ZIim is mainly associated with behaviors such as fear [9, 11], predation [4, 5], and curiosity [12], while ZIil is associated with behaviors such as grooming, chewing, incubation, and jumping [36]. We found that ZIim and ZIil preferentially received inputs from the anterior and posterior MO, which control the movement of different parts of the body [37], and the anterior MO is involved in motor preparation [38]. These results can explain the difference in motor-related behaviors between ZIim and ZIil. Finally, sensory cortex preferentially projected to ZIil and ZIc. ZIc is directly associated with movement and is an important region for deep brain stimulation to treat Parkinson-induced tremor [1, 14]. ZIc preferentially received input from the sensory cortex rather than the MO. ZIc also had bidirectional connection with the reticular nucleus that controls trunk movement and posture. Further, lesions of the ZIc result in the loss of stereotyped movements in rats, while not affecting motivation [39]. Combined their connection with MO, we suggest that the ZIc neurons may be involved in the regulation of stereotyped actions or maintain posture by integrating multisensory information from the cortex. Based on the above information, we speculate that the ZI is involved in emotion and cognition, motivation and motor planning, motor execution and stereotyped actions, and maintaining posture in sequence along the rostro-caudal axis. In addition, cortical neurons that project to the ZI also project to other regions, such as the thalamus, midbrain, and pons [40]. Whether cortical topological output is also present in other regions warrants further investigation.

The projections from ZI to the thalamus were stronger than those from the thalamus. ZI received a lot of input from the cortex, while the output from ZI to the cortex was almost negligible. The thalamus is a "way station" transmitting information to the cerebral cortex [41], and ZI can limit the transmission of ascending sensory information *via* feedforward inhibition of higher order thalamic nuclei [19, 21, 22]. This projection relationship among cortex, ZI, and thalamus is conducive to the cortex to limit the transmission of ascending sensory information *via* ZI. For example, spinal cord injury leads to a significant increase in the activity of PO neurons [8, 42], and this is thought to be caused by a decrease in the activity of GABAergic ZI neurons [43]. And the increased activity of ventral anterior cingulate area (ACAv) neurons increases the activity of GABAergic ZI neurons and significantly improves the symptoms of neuralgia [6, 7]. Therefore, ACAv regulates the intensity of information transmission from PO to cortex by regulating the activity of GABAergic ZI neurons. Once the balance is broken, neuralgia results. In general, the ZI is one of the relay stations through which the cortex regulates thalamic sensory input.

As previously reported, efferent fibers from the ZI were topologically organized in the SC [44]. Moreover, we found that projections from ZI to SC had a longitudinal-zonal organization and the projection of ZI to the lateral SC had a dorsoventral topology, such that the projection of the rostral and caudal ZI to the SC was also topologically organized. Meanwhile, the projection from ZI to PAG was also topological to a certain extent. Neurons of ZIim projected separately to the ventral PAG and ventrolateral SC, which means that ZI can be further divided into more subdomains based on output connectivity to determine its own potential functions. For example, since predation behavior has been reported in the lateral SC and lateral PAG [4, 5, 45], the output from the ZI to the lateral SC may also be associated with predation. It is also worth investigating whether these ZI neurons projecting to the same SC sub-region have the same connecting pattern in other output regions. Analysis of the morphology of single neurons is crucial to understanding the ZI projection patterns.

By applying unbiased cluster analysis, we found that the ZI can be divided into two parts: rostral and intermediomedial (medial part, including ZIr and ZIim), and intermediolateral and caudal (lateral part, including ZIil and ZIc). This may be due to the fact that afferent fibers were rarely limited to a sector of the ZI and the connections of the medial and lateral ZI are significantly different [1, 25]. Unlike the inputs, although the outputs of ZIr and ZIim were also similar, they grouped into two independent clusters. And ZIil and ZIc also had similar properties. The inputs of ZI sectors changed in a progressive manner, and the outputs were organized in a highly topological form. We speculate that similar functions are distributed in adjacent sectors, but participate in different functions through different circuits. We suggest that, similar to the connection results, functions may shift in a progressive manner among different sectors. This also explains why both ZIr and ZIim are involved in different nutritional status-related behaviors, but the binge-eating and predation cycles are strictly restricted to ZIr and ZIim, respectively [5]. There may be a mutual relationship among ZI sectors in the regulation of similar behaviors, which may reflect how the ZI serves as an integrative node for global behavioral modulation [16, 23].

Due to technical constraints, there were limitations that need to be considered. The ZI is formed of a heterogeneous population of cells, all of which play important roles in various functions. We only studied the input and output of GABAergic neurons, but did not investigate the connections of other types of neurons in the ZI. Numerous studies have shown that chemically different neurons may have similar input regions, and that the location of the injection site determines the input pattern. Therefore, our input results are also valuable for studying other types of neuron. The outputs of various types of neuron in the same region may differ. For example, we found that the inputs from the substantia nigra pars reticulata and pallidum were much stronger than the outputs to them. Previous research found that the neurons that project to the basal ganglia are mainly glutamatergic but not GABAergic [46]. Therefore, it is necessary to more fully study the output patterns of chemically different neurons. And whether other types of neuron also have topological connectivity patterns similar to GABAergic neurons is also worth investigating.

We have shown that the connections of the ZI are topologically organized: as the injection site changed, the input-output connection pattern changed significantly, especially the projections from the ZI to the thalamus. Therefore, special attention should be paid to differences in injection sites when comparing different studies. For example, the distribution patterns of efferent fibers from GABAergic ZIr neurons to the SC and PAG that we found, differed from those of Chou *et al.* [16]. This difference is also due to the different injection sites. Chou *et al*. injected the virus more laterally and caudally than we did. The ZIr outputs of Chou *et al*. were more similar to our results for ZIim. They both projected to the lateral SC. The results of Chou *et al*. showed that ZIr (corresponding to our ZIim) had very little projection to the PAG, while our results showed a large number of fibers in the PAG. As we show in Figure 5G and H, ZI neurons projecting to the lateral SC were located near the center. Neurons projecting to the PAG were located closer to the medial ZI. So, we suggest that because their injection site was closer to the central ZI, the neurons projecting to the PAG were not infected. And our results may not uniformly reflect the connectivity strengths of all GABAergic neurons in the ZI. For example, the projection from the ZI to the PO is involved in nocifensive behavior [6], whereas our results showed only a few infected fibers in the dorsal PO. Instead, we found that ZIil has substantial projections to the ventral PO. Neurons projecting to the dorsal PO are located in the region between ZIim and ZIil, and only a few were labeled in this experiment. At the same time, we found that the ZI-to-thalamus projections are strongly topologically organized, but it is difficult to obtain the topological projection patterns by analyzing the projections of populations of neuron. Reconstructing the morphology of individual neurons can avoid the above difficulties and further reveal the organization of ZI projections to the thalamus. Readers should note that we used the signals of fibers here to reflect the strength of the output. Besides that, regions with <0.03% connectivity were set to zero, but that does not mean these regions are not connected with the ZI. Given the technical constraints, we could not directly compare the connectivity strength of ZI sectors, but the data still reflect the connectivity trend of the ZI. Furthermore, it does not affect the conclusion that GABAergic ZI neuronal connectivity is topological. Furthermore, some regions are strongly connected with the ZI, which we do not show in detail. They also have important functions (see supplementary materials). For example, there is a rich connection between the ZI and central amygdala nucleus [47], which plays an important role in fear memory.

In conclusion, we comprehensively mapped the input-output connectivity map of GABAergic ZI neurons that showed topographic organization and preferred input/output circuit connections. Connectivity preference provides a structural basis for understanding complex functions. The topological connection of ZI is important for understanding how the ZI integrates multiple kinds of information and modulates global behaviors.

## Supplementary Information

Below is the link to the electronic supplementary material.

Supplementary file1 (PDF 2998 KB)
