# Supplementary material for: Whole-Brain Connectome of GABAergic Neurons in the Mouse Zona Incerta
Source: Neurosci Bull. 2022 Aug 19;38(11):1315–29. doi: 10.1007/s12264-022-00930-w (PMC9672206; doi:10.1007/s12264-022-00930-w)
Supplement: Supplementary file 1 — Supplementary file1 (PDF 2998 KB) [file 12264_2022_930_MOESM1_ESM.pdf]

Supplemental Figures and Figure Legends

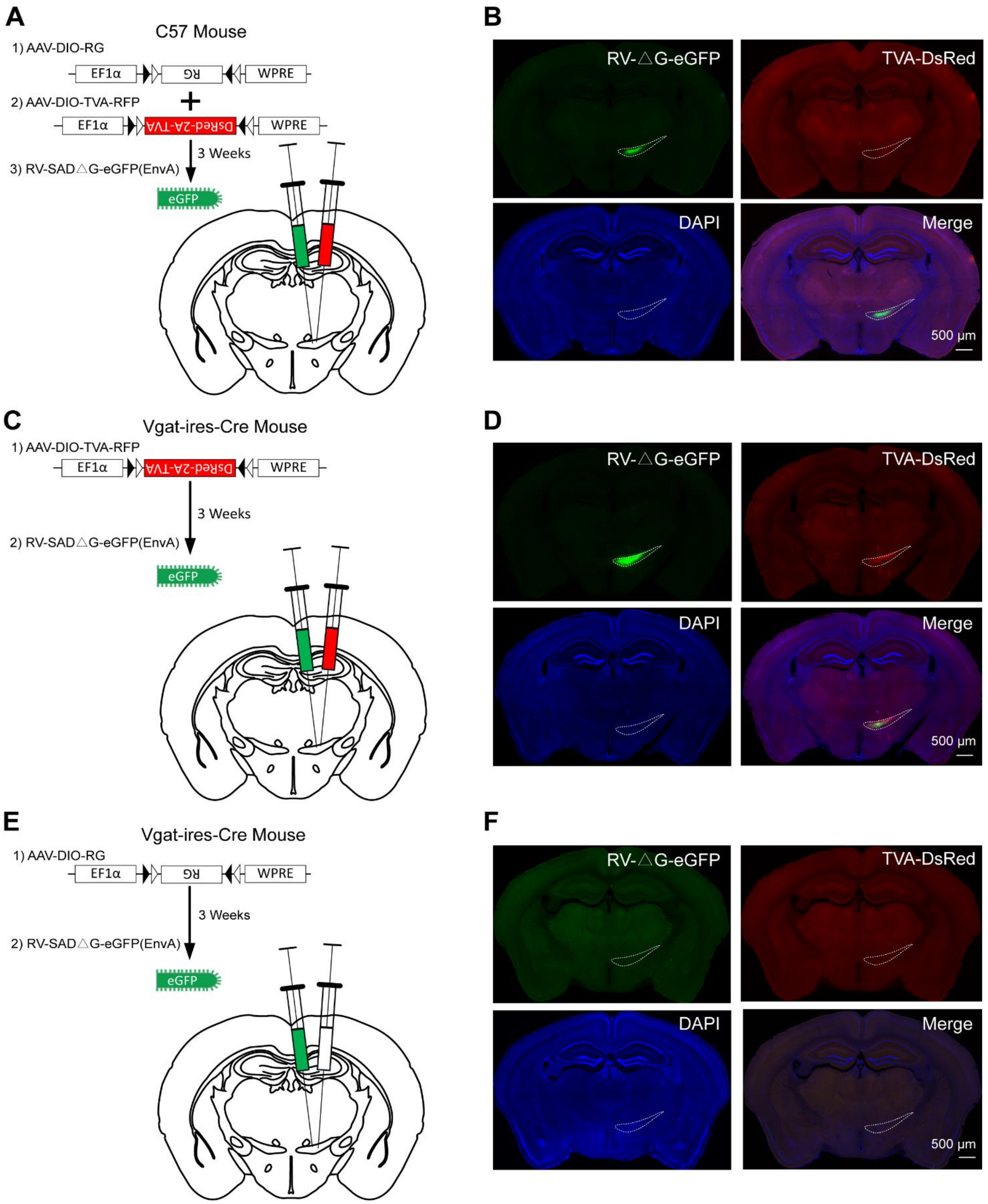

Fig. S1 The RV monosynaptic retrograde tracing strategy is reliable. **A** AAV9-EF1α-DIO-RG (1) and AAV9-EF1α-DIO-DsRed-TVA (2) is injected into wild-type mice, and three weeks later RV-SAD-

△G-eGFP (EnvA) (3) is injected into the same ZI sector. **B** eGFP-positive cells in the ZI are all near the injection site. This verifies that the monosynaptic retrograde tracing strategy is Cre-dependent. Scale bar, 500 μm. **C** AAV9-EF1α-DIO-DsRed -TVA (1) is injected into Vgat-Cre mice, and three weeks later RV-SAD-△G-eGFP (EnvA) (2) is injected into the same ZI sector. **D** eGFP- and DsRed-positive cells in ZI, and no eGFP-positive cells outside the injection site. This verifies that the monosynaptic retrograde tracing strategy is RG-dependent. Without RG, RV could not spread retrogradely to presynaptic neurons. Scale bar, 500 μm. **E** AAV9-EF1α-DIO-RG (1) is injected into Vgat-Cre mice, and three weeks later RV-SAD-△G-eGFP (EnvA) (2) is injected into the same ZI sector. **F** No eGFP- or DsRed-positive cells in the whole brain, verifying that the monosynaptic retrograde tracing strategy is TVA-dependent. Modified RV only infects TVA-positive neurons. Scale bar, 500 μm.

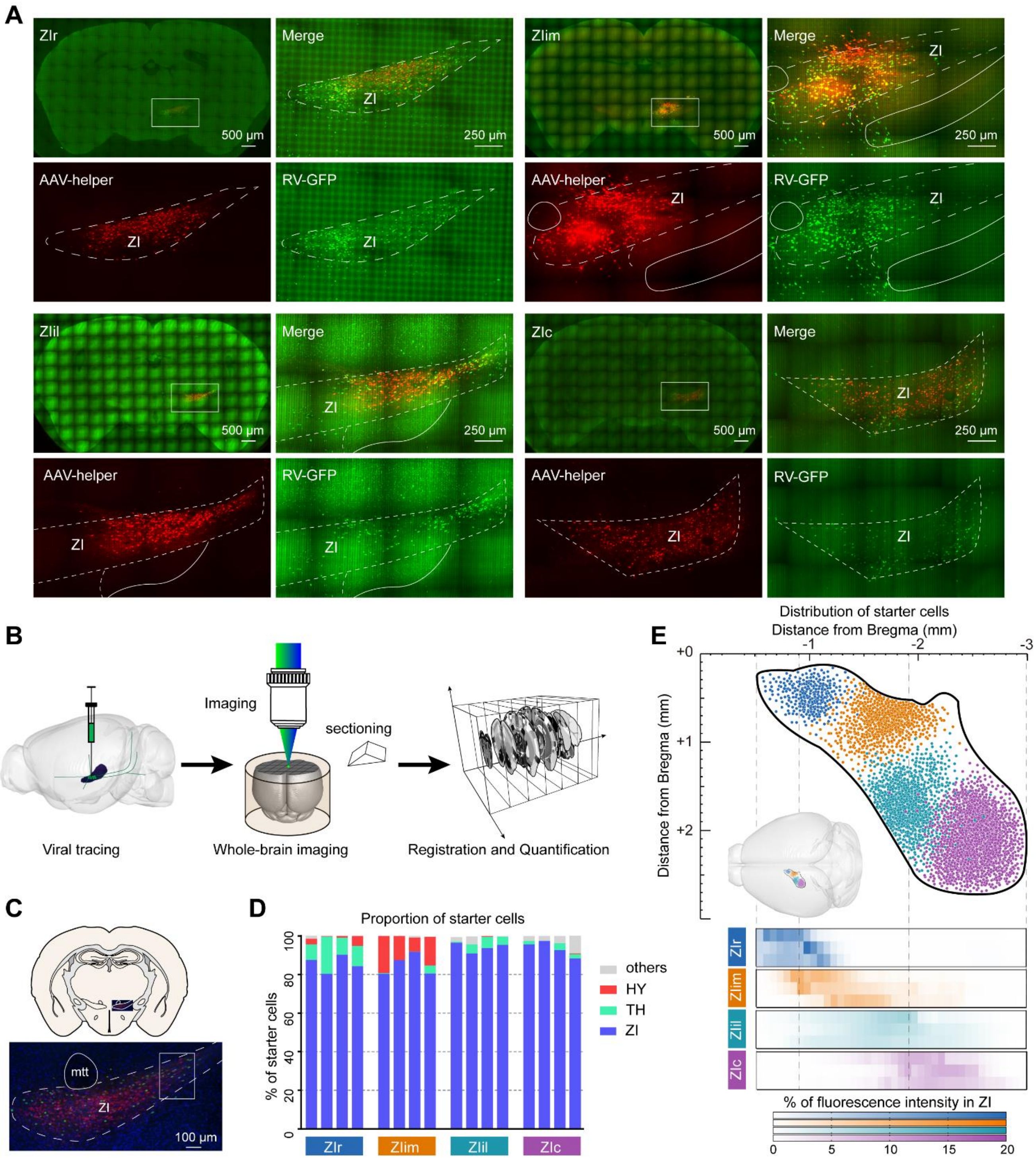

Fig. S2 Workflow and location of starter cells. **A** Distribution of starter cells in different ZI sectors in the input strategy. Scale bars, 500 μm (inset) and 250 μm (others). **B** Workflow of viral tracing, whole-brain imaging, registration, and quantification. **C** Location of the starter cells in the ZI. **D** Proportions of labeled starter neurons in injection regions (100%, all detected starter cells). Scale bar, 100 μm. **E** Schematic of the top view of anterograde tracing starter cells in distinct ZI sectors and heat map showing the fluorescence intensity distribution in each sample at each specific ZI target (lower panels). The density and brightness of injection regions are too high to distinguish the cell bodies. Since the

fluorescence brightness of neuron cell bodies was much greater than that of efferent axons, the distribution of fluorescence brightness was used to show the distribution of infected neurons in the ZI. Center left insert, visual aid representation the location of the ZI. Every row in each ZI sector is from one sample, *n* = 3 mice per condition in the anterograde strategy.

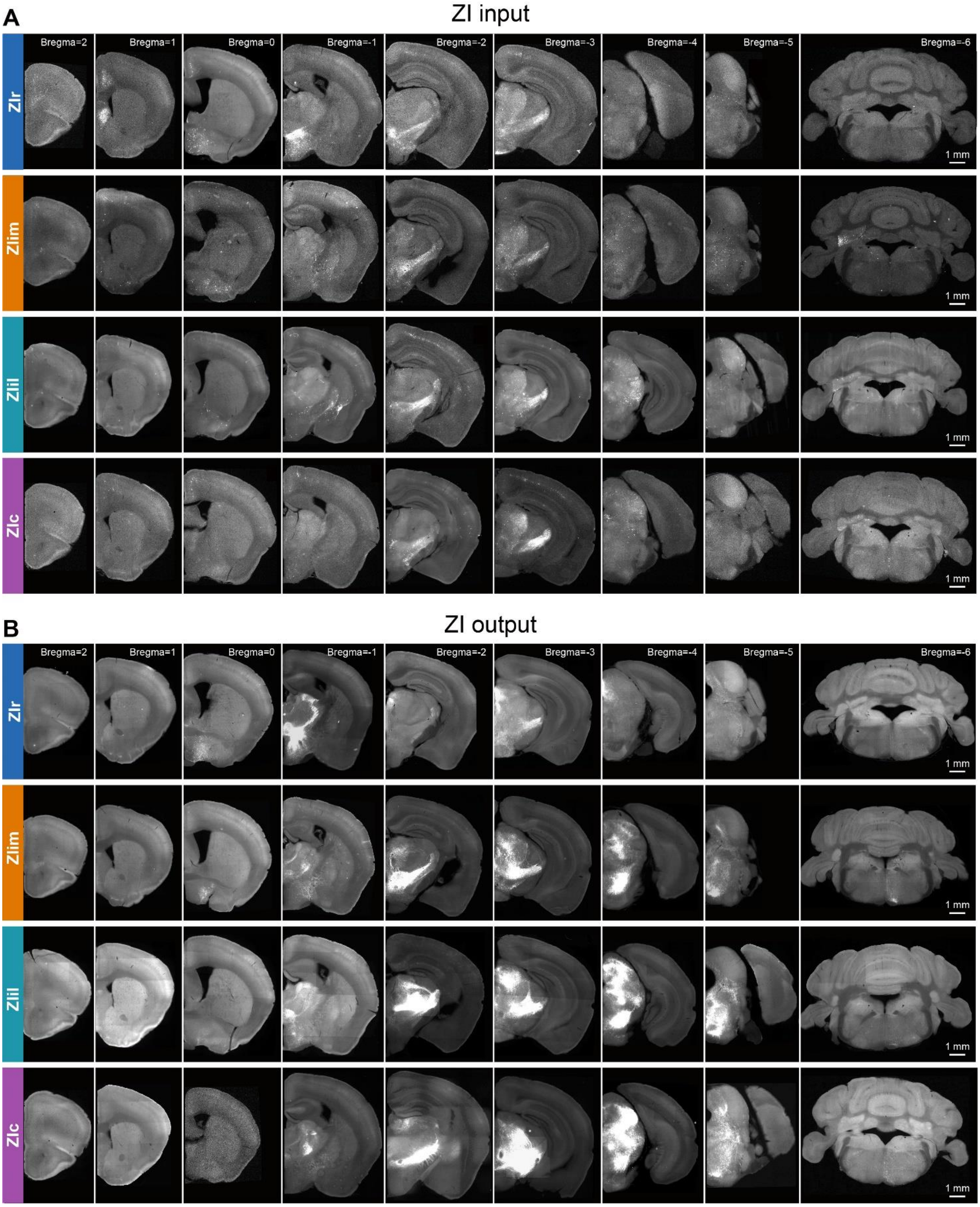

Fig. S3 Representative images of ZI sector inputs and outputs. **A** Continuous coronal view of ZI sector inputs. **B** Continuous coronal view of ZI sector outputs. Slice thickness, 100 μm. Scale bars, 1 mm.

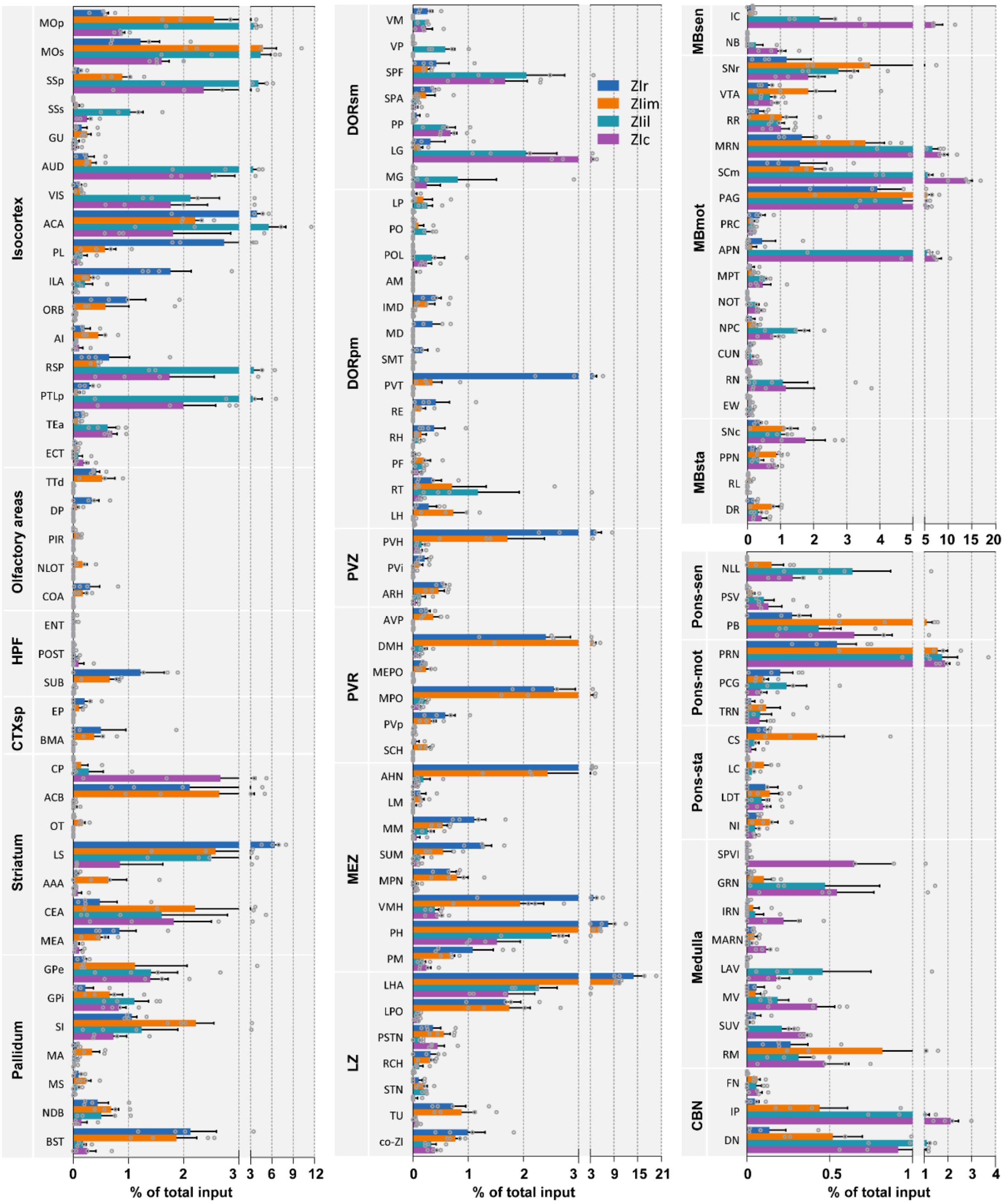

Fig. S4 Brain-wide input datasets for ZIr, ZIim, ZIil, and ZIc**.** Whole-brain input regions are divided into 126 subregions for comparison. Values are presented as normalized percentage of total cells (RV). Data shown as the mean ± SEM. n = 4 mice per condition.

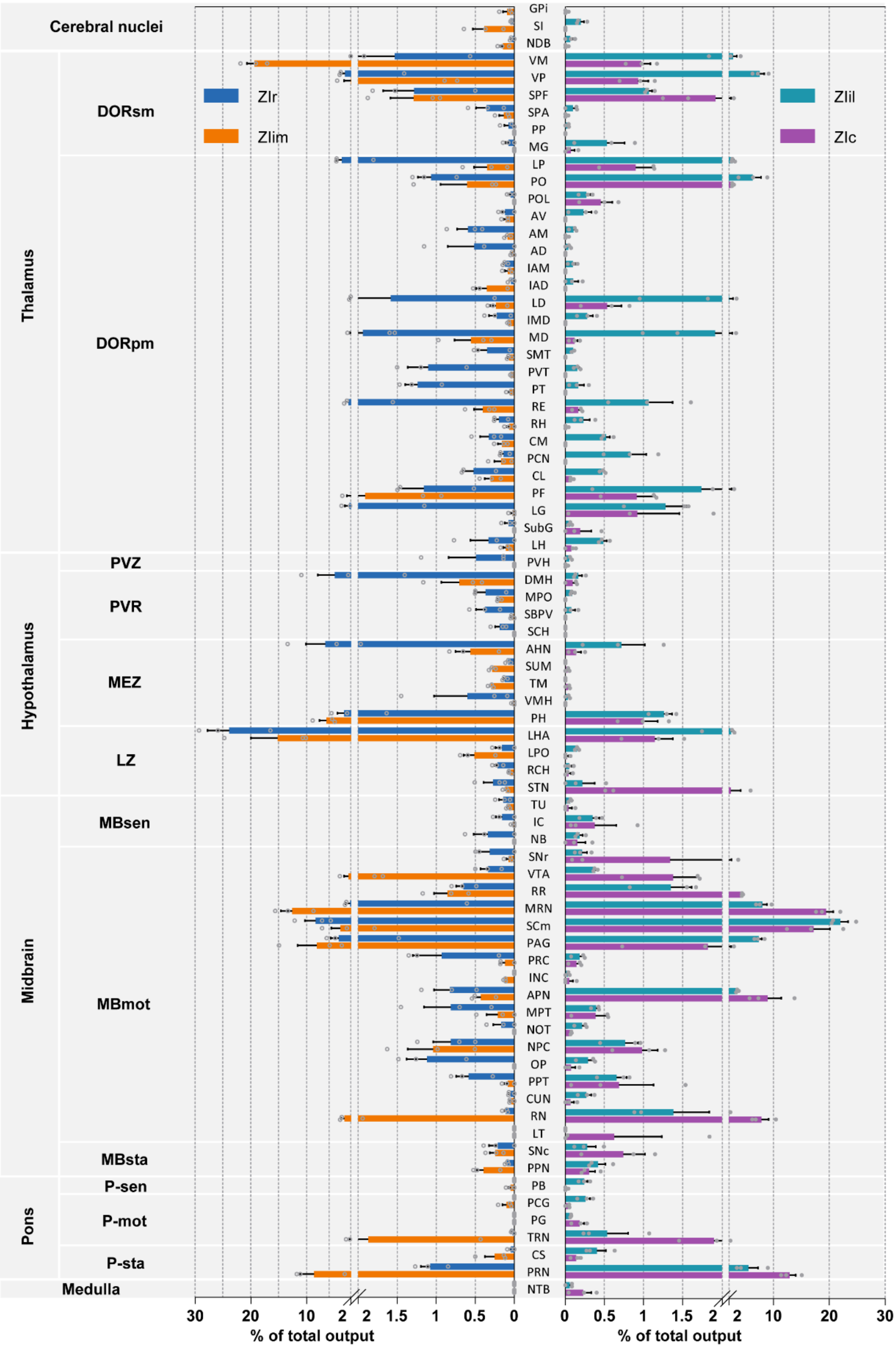

Fig. S5 Brain-wide output datasets for ZIr, ZIim, ZIil, and ZIc**.** Whole-brain output regions are divided into 77 subregions for comparison. Values are presented as normalized percentage of total pixels (AAV). Data shown as the mean ± SEM. n = 3 mice per condition.

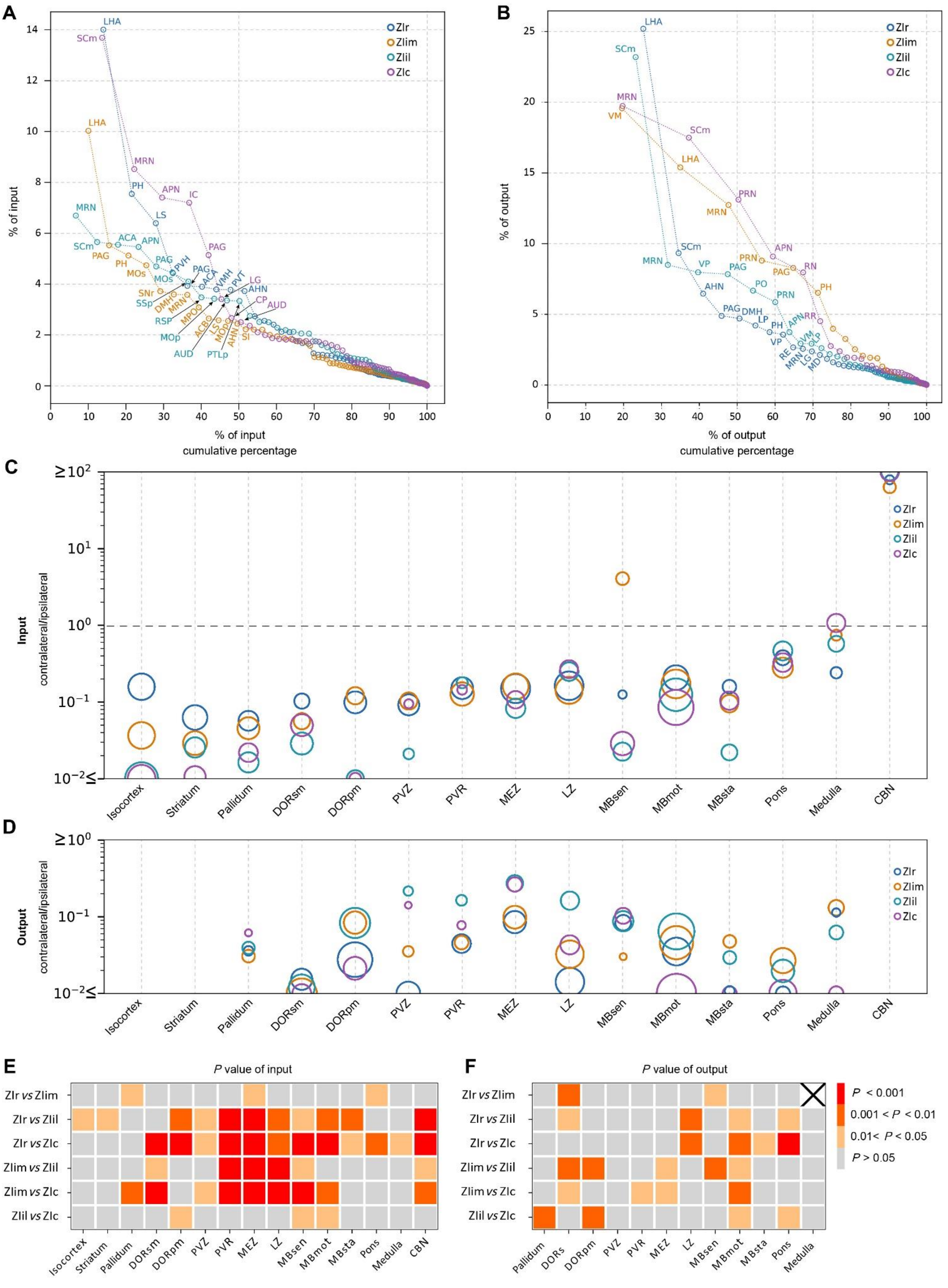

Fig. S6 Characteristics of ZI connectivity. **A** Cumulative input percentage, in the order of largest to

smallest. **B** Cumulative output percentage, in the order of largest to smallest (horizontal axis, cumulative percentage; vertical axis, average value of input strength of a single region). **C** Ipsilateral and contralateral input ratios. **D** Ipsilateral and contralateral output ratios. Area of a circle indicates strength of connection. **E** Input p-value heat map between the four ZI sectors. **F** Output p-value heat map between the four ZI sectors. Input data, *n* = 4 mice per condition in **A**, **C**, and **E**. Output data, *n* = 3 mice per condition in **B**, **D**, and **F**. Colors indicate data from each ZI sector: ZIr, blue; ZIim, orange; ZIil, cyan; ZIc, purple.

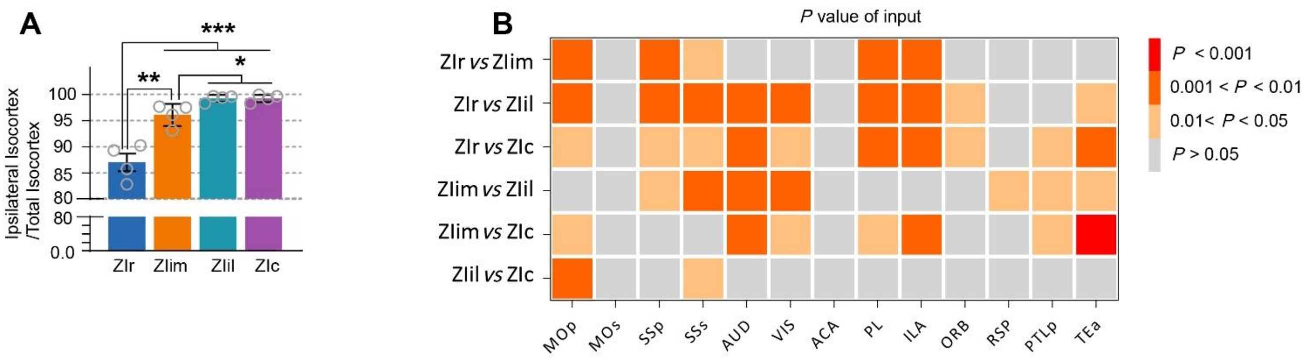

Fig. S7 Characteristics of isocortex projection to ZI. **A** Ratio of the number of eGFP-positive neurons in ipsilateral isocortex to the number of eGFP-positive neurons in the whole isocortex (100% indicates all cortical eGFP-positive cells). **B** Isocortex input p-value heat map between the four ZI sectors. *n* = 4 mice per condition in **A** and **B**.

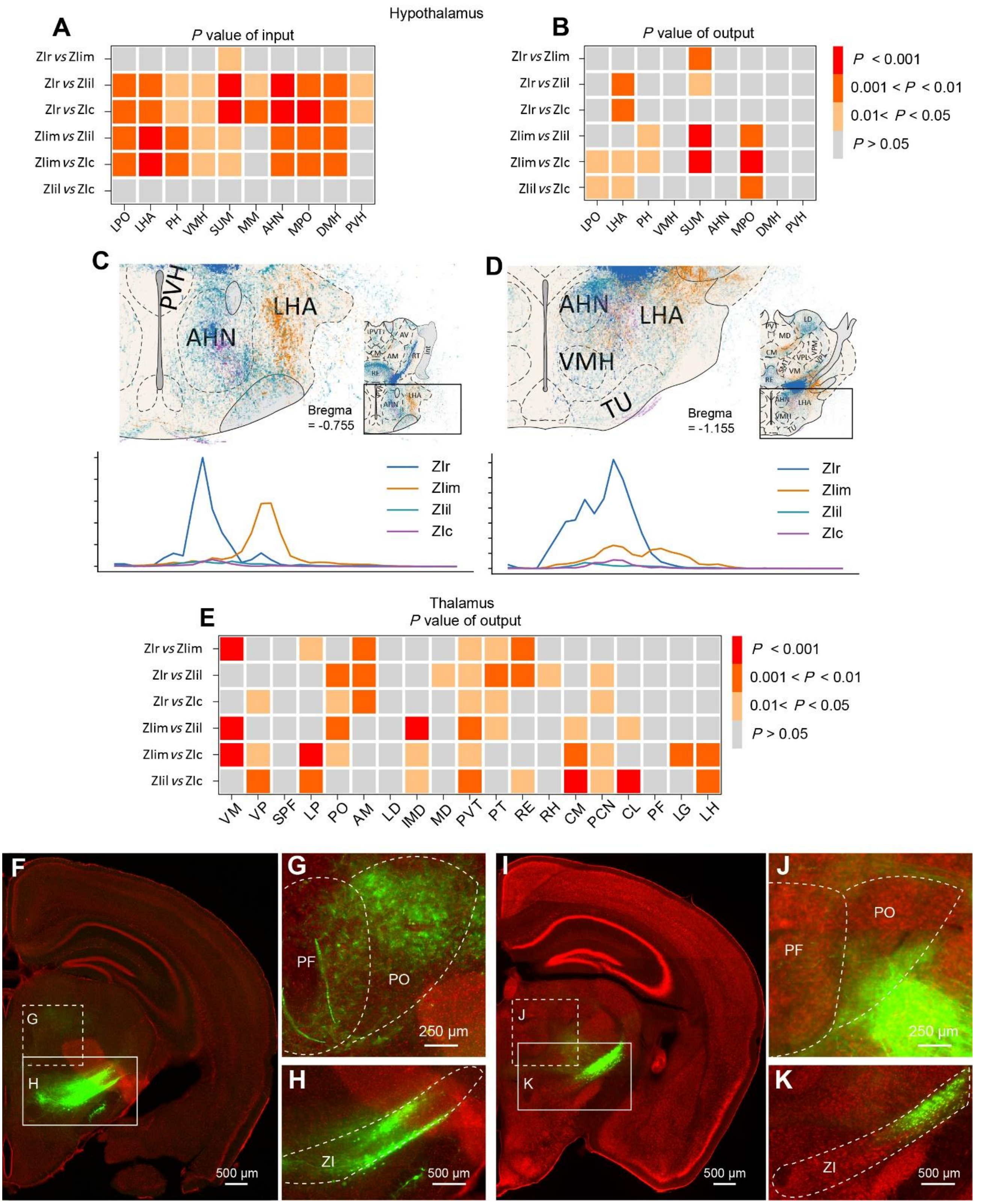

Fig. S8 Characteristics of ZI connectivity. **A** Hypothalamic input p-value heat map between the four ZI sectors. **B** Hypothalamic output p-value heat map between the four ZI sectors. **C**, **D** Schematic coronal sections depicting the distribution of the eGFP signal in the hypothalamus (eGFP signals represent the output of ZI sectors; lower curves, output strengths to the hypothalamus; bin width, 100

μm). **E** Thalamic output p-value heat map between the four ZI sectors. **F** Output from ZIi center neuron to the dorsal PO. **G**, **H** Enlarged view of the area indicated by the rectangle in **F**. **G** distribution of fibers in the PO, **H** distribution of somata in ZI. **I** Outputs from ZIil neurons to the ventral PO. **J**, **K** enlarged view of the area indicated by the rectangle in **I**. **J** distribution of fibers in the PO, **K** distribution of somata in the ZI. $n = 4$ mice per condition in **A**. $n = 3$ mice per condition in **B** and **E**. Slice thickness, 100 μm in **C**, **D**, and **F**–**K**). Scale bars, 500 μm (**F**, **H**, **I**, and **K**) and 250 μm (**G** and **J**).

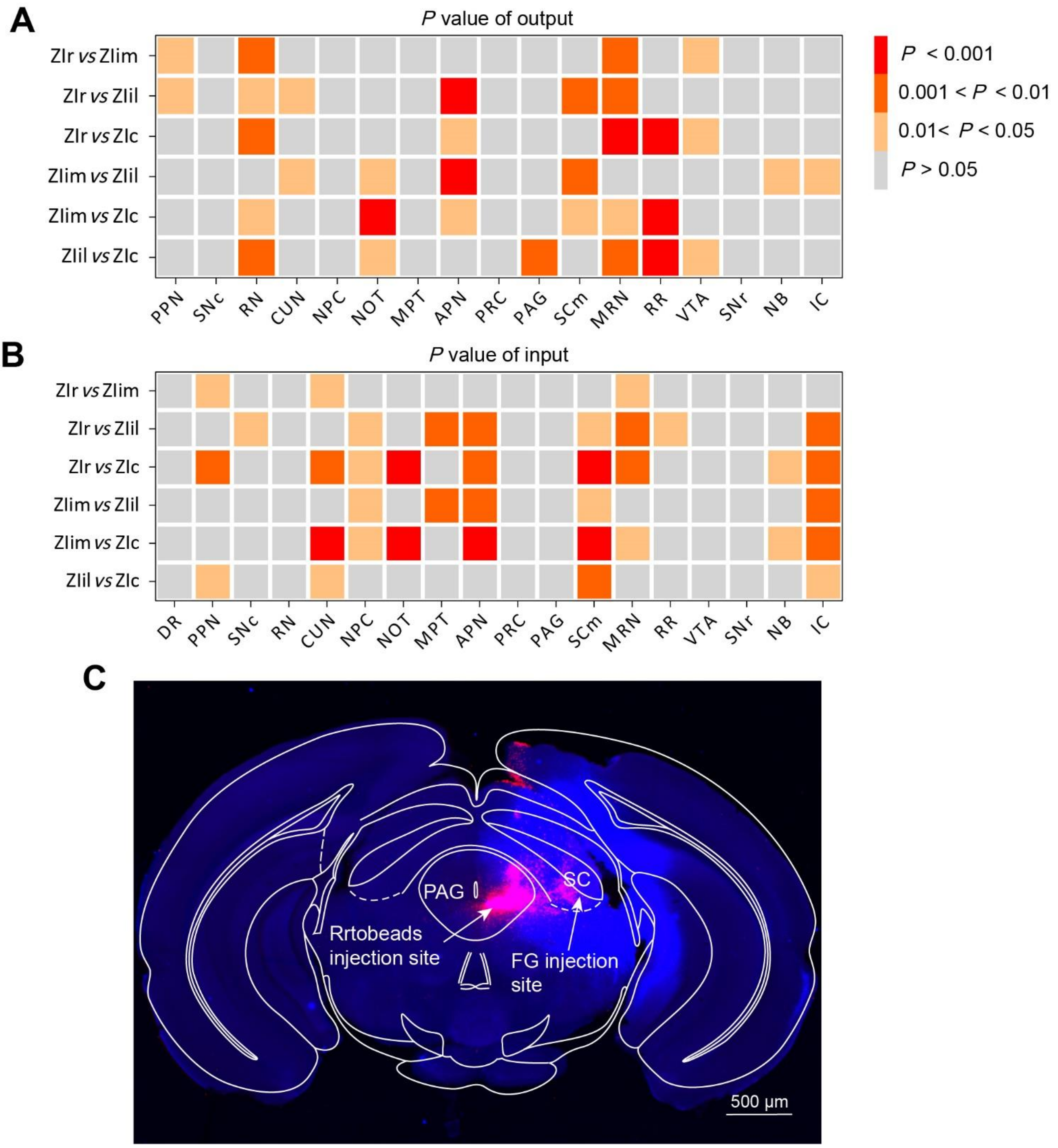

Fig. S9 Characteristics of connections between ZI and midbrain. **A** Midbrain input p-value heat map between the four ZI sectors (*n* = 3 mice per condition). **B** Midbrain output p-value heat map between the four ZI sectors (*n* = 4 mice per condition). **C** Coronal section showing the injection sites of retrobeads and FG. Scale bar, 500 μm.

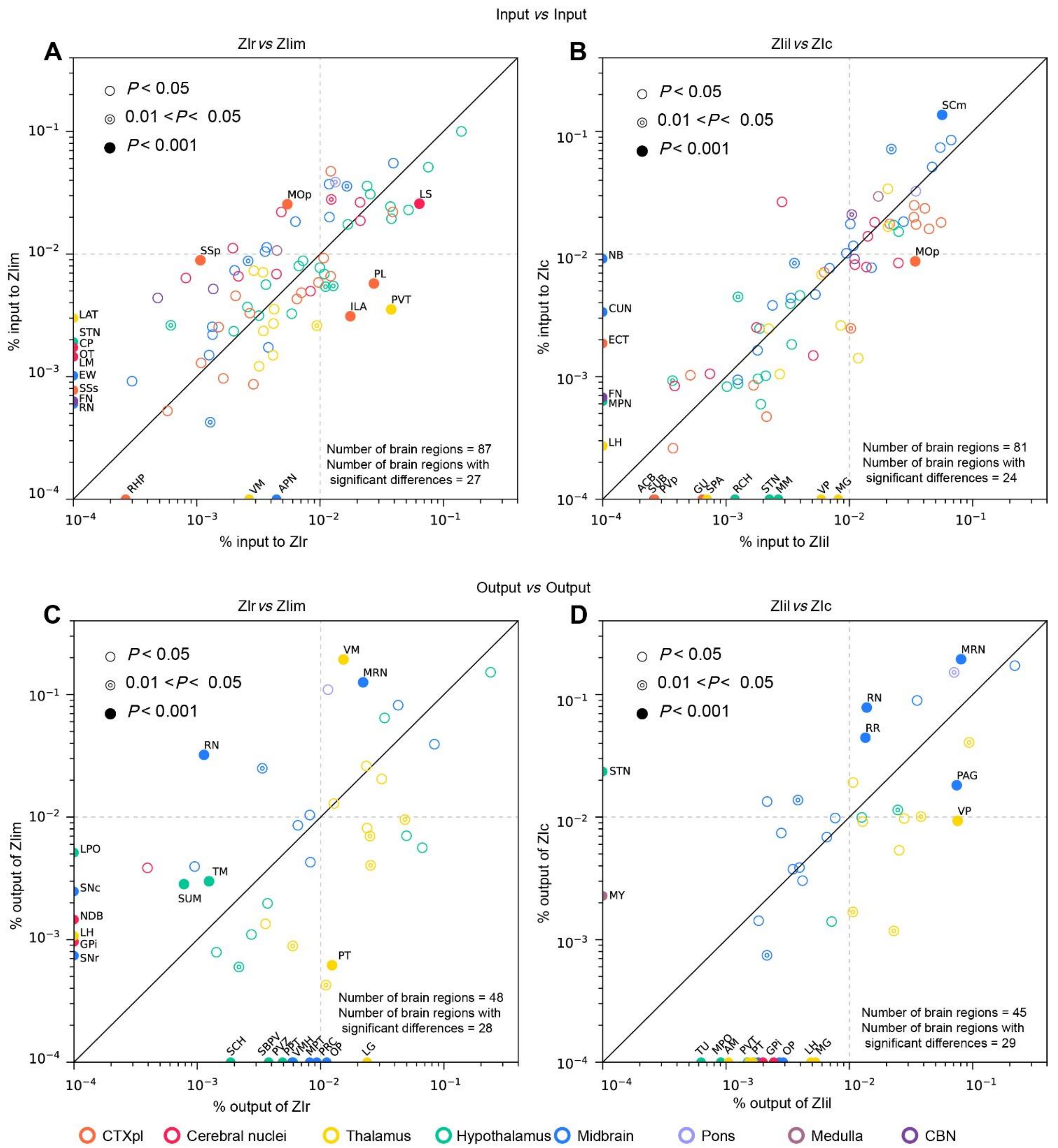

Fig. S10 Comparison of connection patterns between ZI sectors**. A** Pattern between ZIr input and ZIim input. **B** Pattern between ZIil input and ZIc input. Horizontal and vertical axes represent the average values of the inputs in **A** and **B**. **C** Pattern between ZIr output and ZIim output. **D** Pattern between ZIil output and ZIc output. The color of circles represents the main regions to which these circles belong (see illustration below). Circles (○, *P* >0.05) indicate no significant difference between inputs and outputs, while concentric circles (◎, 0.01 < *P* < 0.05) and solid circles (●, *P* <0.01) indicate a statistical difference between inputs and outputs. Lower right, number of regions and the number of regions with

significant differences between ZI sectors. Points on the horizontal axis represent regions with very little output (<0.03%), and points on the vertical axis represent regions with very little input (<0.03%). The difference in outputs between ZIr and ZIim is larger than the inputs; similarly for ZIil and ZIc.

Abbreviations

AAA	Anterior amygdala area	MS	Medial septal nucleus
ACA	Anterior cingulate area	MV	Medial vestibular nucleus
ACB	Nucleus accumbens	NB	Nucleus of the brachium of the inferior colliculus
AD	Anterodorsal nucleus	NDB	Diagonal band nucleus
AHN	Anterior hypothalamic nucleus	NI	Nucleus insertus
AI	Agranular insular area	NLL	Nucleus of the lateral lemniscus
AM	Anteromedial nucleus	NOT	Nucleus of the optic tract
APN	Anterior pretectal nucleus	NPC	Nucleus of the posterior commissure
ARH	Arcuate hypothalamus nucleus	NTB	Nucleus of the trigeminal body
AUD	Auditory areas	OP	Olivary pretectal nucleus
AV	Anteroventral nucleus of thalamus	ORB	Orbital area
AVP	Anteroventral periventricular nucleus	OT	Olfactory tubercle
BMA	Basomedial amygdala nucleus	PAG	Periaqueductal gray
BST	Bed nuclei of the stria terminalis	PB	Parabrachial nucleus
CBN	Cerebellar nuclei	PCG	Pontine central gray
CEA	Central amygdala nucleus	PCN	Paracentral nucleus
CL	Central lateral nucleus of the thalamus	PF	Parafascicular nucleus
CM	Central medial nucleus of the thalamus	PG	Pontine gray
CP	Caudatoputamen	PH	Posterior hypothalamic nucleus
CS	Superior central nucleus raphe	PL	Prelimbic area
CTXpl	Cortical plate	PM	Premammillary nucleus

CTXsp	Cortical subplate	PO	Posterior complex of the thalamus
CUN	Cuneiform nucleus	POL	Posterior limiting nucleus of the thalamus
DMH	Dorsomedial nucleus of the hypothalamus	POST	Postsubiculum
DN	Dentate nucleus	PP	Peripeduncular nucleus
DORpm	Thalamus, polymodal association cortex related	PPN	Pedunculopontine nucleus
DORsm	Thalamus, sensory-motor cortex related	PPT	Posterior pretectal nucleus
DR	Dorsal raphe nucleus	PRC	Precommissural nucleus
ECT	Ectorhinal area	PRN	Pontine reticular nucleus
ENT	Entorhinal area	PSTN	Parasubthalamic nucleus
EP	Endopiriform nucleus	PSV	Principal sensory nucleus of the trigeminal
EW	Edinger-Westphal nucleus	PT	Parataenial nucleus
FN	Fastigial nucleus	PTLp	Posterior parietal association areas
GPe	Globus pallidus, external segment	PVH	Parventricular hypothalamic nucleus
GPi	Globus pallidus, internal segment	PVi	Periventricular hypothalamic nucleus, intermediate part
GRN	Gigantocellular reticular nucleus	PVp	Periventricular hypothalamic nucleus, posterior part
GU	Gustatory area	PVR	Periventricular region
HPF	Hippocampal formation	PVT	Paraventricular nucleus of the thalamus
IAD	Interanterodorsal nucleus of the thalamus	PVZ	Periventricular zone
IAM	Interanteromedial nucleus of the thalamus	RCH	Retrochiasmatic area
IC	Inferior colliculus	RE	Nucleus reuniens
ILA	Infralimbic area	RH	Rhomboid nucleus

IMD	Intermediodorsal nucleus of the thalamus
INC	Interstitial nucleus of Cajal
IP	Interposed nucleus
IRN	Intermediate reticular nucleus
LAT	Lateral group of dorsal thalamus
LAV	Lateral vestibular nucleus
LC	Locus ceruleus
LD	Lateral dorsal nucleus of thalamus
LDT	Laterodorsal tegmental nucleus
LG	Lateral geniculate complex
LHb	Lateral habenula
LHA	Lateral hypothalamic area
LM	Lateral mammillary nucleus
LP	Lateral posterior nucleus of the thalamus
LPO	Lateral preoptic area
LS	Lateral septal nucleus
LT	Lateral terminal nucleus of the accessory optic tract
LZ	Hypothalamus lateral zone
MA	Magnocellular nucleus
MARN	Magnocellular reticular nucleus
MBmot	Midbrain, motor related
MBsen	Midbrain, sensory related
MBsta	Midbrain, behavior related
MD	Mediodorsal nucleus of the thalamus
MEA	Medial amygdala nucleus
MEPO	Median preoptic nucleus
MEZ	Hypothalamus medial zone

RHP	Retrohippocampal region
RL	Rostral linear nucleus raphe
RM	Nucleus raphe magnus
RN	Red nucleus
RR	Midbrain reticular nucleus, retrorubral area
RSP	Retrosplenial area
RT	reticular nuclei of dorsal thalamus
SBPV	Subparaventricular zone
SCH	Subrachiasmatic nucleus
SCm	Superior colliculus, motor related
SI	Substantia innominata
SMT	Submedial nucleus of the thalamus
SNc	Substantia nigra, pars compacta
SNr	Substantia nigra, pars reticulata
SPA	Subparafascicular area
SPF	Subparafascicular nucleus
SPVI	Spinal nucleus of the trigeminal, interpolar part
SSp	Primary somatosensory areas
SSs	Supplemental somatosensory area
STN	Subthalamic nucleus
SUB	Subiculum
SubG	Subgeniculate nucleus
SUM	Supramammillary nucleus
SUV	Superior vestibular nucleus
TEa	Temporal association areas
TM	Tuberomammillary nucleus
TRN	Tegmental reticular nucleus

MG	Medial geniculate complex	TU	Tuberal nucleus
MM	Medial mammillary nucleus	VIS	Visual areas
MOp	Primary motor area	VM	Ventral medial nucleus of the thalamus
MOs	Secondary motor area	VMH	Ventromedial hypothalamic nucleus
MPN	Medial preoptic nucleus	VP	Ventral posterior complex of the thalamus
MPO	Medial preoptic area	VTA	Ventral tegmental area
MPT	Medial pretectal area	ZI	Zona incerta
MRN	Midbrain reticular nucleus	co-ZI	Contralateral ZI
